# Pharmacological strategies to overcome immune checkpoint inhibitor resistance in non-small cell lung cancer

**DOI:** 10.3389/fonc.2025.1665239

**Published:** 2026-01-08

**Authors:** Yuyu Xu, Huiling Shen, Dong Shang, Cailin Zhu

**Affiliations:** 1 Department of Oncology, Zhenjiang First People's Hospital, Zhenjiang, China; 2 Department of Respiratory and Critical Care Medicine, The First Affiliated Hospital of Xi'an Jiaotong University, Xi’an, China; 3 Department of Thoracic Surgery, the First Affiliated Hospital of Xi’an Jiaotong University, Xi’an, China

**Keywords:** epigenetic modulation, immune checkpoint inhibitors (ICIs), immunotherapy resistance, non-small cell lung cancer (NSCLC), PD-1/PD-L1 blockade, tumor microenvironment (TME)

## Abstract

Immune checkpoint inhibitors (ICIs) have redefined the therapeutic paradigm of non-small cell lung cancer (NSCLC), offering durable remission in select patients by reactivating anti-tumor T cell responses. Yet, this clinical triumph is tempered by the reality that most patients experience either primary resistance or relapse due to acquired resistance, underscoring an urgent need for mechanistically grounded solutions. Resistance arises through a complex interplay of tumor-intrinsic mechanisms, including defects in antigen presentation, interferon signaling disruption, and oncogenic pathway activation (EGFR, KRAS, MET), and tumor-extrinsic factors such as immunosuppressive cell populations, inhibitory cytokines, and metabolic rewiring of the tumor microenvironment (TME). This review provides a comprehensive synthesis of emerging pharmacological strategies aimed at reversing ICI resistance in NSCLC. Promising avenues include dual or multi-checkpoint inhibition (targeting LAG-3, TIGIT, TIM-3), integration of epigenetic reprogrammers to resensitize immune-silent tumors, and metabolic interventions that normalize the TME. Additionally, combination regimens with oncogene-directed therapies, engineered cytokine analogs, neoantigen-based vaccines, and adoptive T cell therapies are reshaping the frontier of immunoresistant NSCLC management. We also highlight pivotal clinical trials—both completed and ongoing that illuminate translational breakthroughs and therapeutic pitfalls. Looking ahead, the field must grapple with key challenges: the refinement of predictive biomarkers, stratification of patients through genomic, immunologic, and microbiome-based profiling, and the management of toxicity in complex combination protocols. Ultimately, a shift toward highly personalized, biomarker-guided therapeutic strategies holds the greatest promise for overcoming resistance and extending the reach of immunotherapy in NSCLC.

## 1 Introduction

Lung cancer accounts for approximately 2.5 million new diagnoses worldwide in 2022 and was responsible for more than 1.8 million deaths in the same year (1). A primary challenge in managing NSCLC is its frequent diagnosis at an advanced stage, which significantly limits therapeutic options. Consequently, the five-year survival rate for patients with advanced NSCLC remains below 20%, reflecting the ongoing struggle to achieve durable responses with current therapeutic strategies (2–5). NSCLC is characterized by significant molecular heterogeneity, and its classification is further refined based on histopathological and genetic criteria. The disease is broadly categorized into three major histological subtypes: adenocarcinoma, squamous cell carcinoma, and large cell carcinoma, each with distinct morphological features and underlying molecular alterations that influence disease progression and therapeutic responsiveness (6–8). Advances in molecular profiling have led to the identification of key driver mutations in genes such as EGFR, ALK, KRAS, ROS1, and MET, which have enabled the development of targeted therapeutic agents tailored to these oncogenic alterations. However, the clinical utility of targeted therapies remains limited by two major challenges: the emergence of therapeutic resistance and the low prevalence of actionable mutations across the broader NSCLC patient population. These limitations underscore the urgent need for novel and more universally applicable treatment strategies (9, 10).

Over the last decade, the introduction of immune checkpoint inhibitors (ICIs) has significantly transformed the therapeutic landscape of NSCLC. These agents function by reactivating the immune system’s capacity to detect and eliminate malignant cells, which frequently escape immune surveillance through the exploitation of inhibitory checkpoint pathways. In NSCLC, the most extensively targeted immune checkpoints are cytotoxic T lymphocyte-associated antigen 4 (CTLA-4) and the programmed death-1 (PD-1)/programmed death-ligand 1 (PD-L1) axis, both of which play pivotal roles in regulating T cell-mediated antitumor responses (11).

The PD-1 receptor is an inhibitory molecule expressed on T lymphocytes that, upon binding to its ligand PD-L1, commonly upregulated on tumor cells, suppresses T cell proliferation and reduces cytokine secretion. Therapeutic agents targeting this pathway, such as PD-1 inhibitors (nivolumab, pembrolizumab) and PD-L1 inhibitors (atezolizumab), disrupt this interaction and thereby reinvigorate antitumor immune responses. In contrast, CTLA-4 acts earlier in the immune activation cascade, primarily within lymphoid tissues. It competes with the co-stimulatory receptor CD28 for engagement with B7 molecules on antigen-presenting cells, thereby attenuating T cell activation. Inhibitors of CTLA-4, including ipilimumab, enhance T cell priming and expansion by lifting this inhibitory checkpoint, promoting more robust immune activation (12). Multiple landmark clinical trials, including KEYNOTE-024, KEYNOTE-189, CheckMate 227 (13, 14), and IMpower110, have firmly established ICIs as a cornerstone of NSCLC therapy. These studies demonstrated significant overall survival improvement in both adenocarcinoma and squamous subtypes, particularly among patients with high PD-L1 expression or elevated tumor mutational burden. As a result, ICIs have become a cornerstone of first-line therapy in advanced NSCLC, administered either as monotherapy or in combination with other therapeutic agents depending on biomarker status and disease characteristics (15).

**Figure 1:**
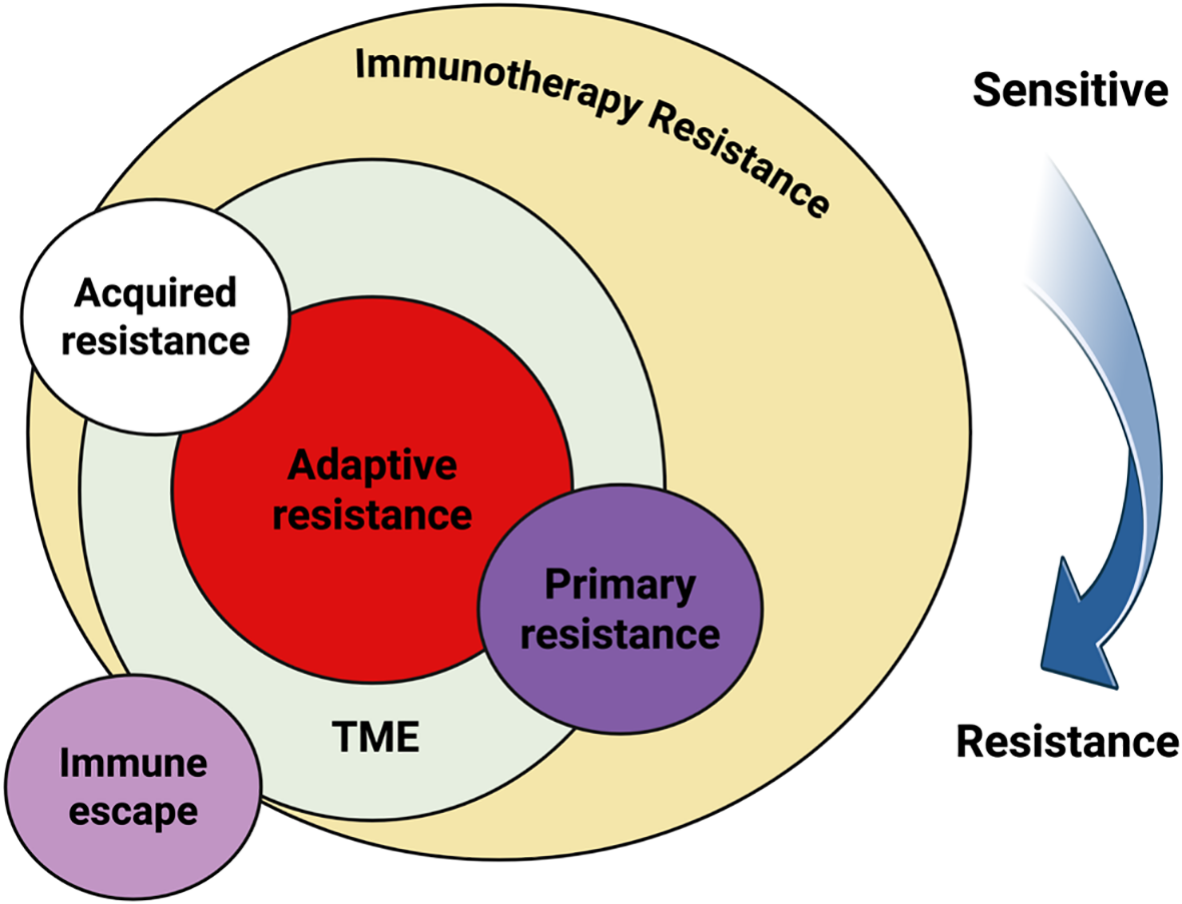
The interplay among primary resistance, acquired resistance, adaptive resistance, and immune escape during immunotherapy can be understood through their underlying mechanisms and clinical manifestations. Both primary and acquired resistance stem from alterations in tumor cells as well as changes within the TME. In contrast, adaptive resistance is confined exclusively to modifications within the TME. Clinically, adaptive resistance presents with features indistinguishable from those observed in primary or acquired resistance (288). Although immune escape culminates in outcomes similar to drug resistance, it differs fundamentally because the resistant tumor clones lack the specific molecular targets of therapy; therefore, immune escape should not be classified as genuine drug resistance. The progression of the immune response from a sensitive state to resistance captured by the blue arrow parallels the development of both primary and acquired resistance in the context of immunotherapy.

Despite the substantial advancements brought by ICIs, their clinical benefit is not universal. A considerable subset of patients exhibits primary resistance, characterized by an absence of therapeutic response from the outset. Others may initially respond but subsequently develop acquired resistance during the early stages of treatment. The mechanisms underlying both forms of resistance are complex and multifactorial, involving a combination of tumor-intrinsic factors such as genetic mutations and altered signaling pathways and immune-related mechanisms, including impaired antigen presentation, T cell exclusion, or the presence of immunosuppressive cells within the tumor microenvironment. Understanding these resistance pathways remains a critical challenge in optimizing immunotherapy outcomes for NSCLC (16). Key contributors to Immune checkpoint inhibitor (ICI) resistance include disruptions in the antigen presentation machinery, such as mutations or loss of β2-microglobulin (β2M), alongside impaired interferon signaling pathways and diminished neoantigen expression, all of which hinder effective immune recognition. Additionally, the presence of a highly immunosuppressive Tumor microenvironment (TME), enriched with regulatory T cells (Tregs), myeloid-derived suppressor cells (MDSCs), and the upregulation of alternative inhibitory receptors like LAG-3, TIM-3, and TIGIT, further impedes anti-tumor immunity. Addressing these resistance mechanisms is essential for enhancing therapeutic efficacy and improving clinical outcomes in patients with NSCLC (17). **Figure 1** delineates the mechanisms of primary, acquired, and adaptive resistance, as well as immune escape, illustrating their clinical manifestations and impact on immunotherapy outcomes in NSCLC.

This review aims to explore and categorize emerging pharmacological strategies designed to overcome ICI resistance in NSCLC. Strategies under investigation include combination therapies with other immune modulators (e.g., LAG-3, TIGIT inhibitors), targeted therapies (e.g., EGFR or KRAS inhibitors), and epigenetic modulators that reprogram the tumor microenvironment to be more immunogenic. In addition, novel agents such as STING agonists, oncolytic viruses, and personalized cancer vaccines are being evaluated in preclinical and clinical settings (18, 19). By systematically examining these approaches, the objective is to provide a comprehensive overview of how pharmacological innovation may help surmount the challenge of ICI resistance in NSCLC and ultimately improve patient outcomes.

## 2 Mechanisms of resistance to immune checkpoint inhibitors

### 2.1 Primary vs. acquired resistance: definitions and classification

Resistance to ICIs can be broadly categorized into primary and acquired forms.

Primary resistance refers to the lack of a meaningful clinical response at the onset of therapy, whereas acquired resistance is defined as tumor progression after an initial period of response. Clinically, primary resistance is typically observed within the first 8–12 weeks of treatment, while acquired resistance usually emerges after a sustained disease control lasting at least six months (20–22). Effective ICI therapy relies on the reactivation of T cells that recognize tumor-specific neoantigens; however, deficiencies in antigen processing or presentation, along with a lack of immunogenic neoantigens, are closely linked to impaired anti-tumor immune responses (22–25). Primary resistance is often driven by tumor-intrinsic mechanisms such as defective antigen presentation, loss of interferon signaling, or oncogenic pathway activation that prevent effective immune priming. Although similar resistance mechanisms are observed across various solid tumors, recent evidence highlights that NSCLC exhibits unique resistance signatures shaped by its mutational landscape, tumor microenvironment, and exposure to smoking-related carcinogens.

#### 2.1.1 Tumor-intrinsic mechanisms

**Figure 2:**
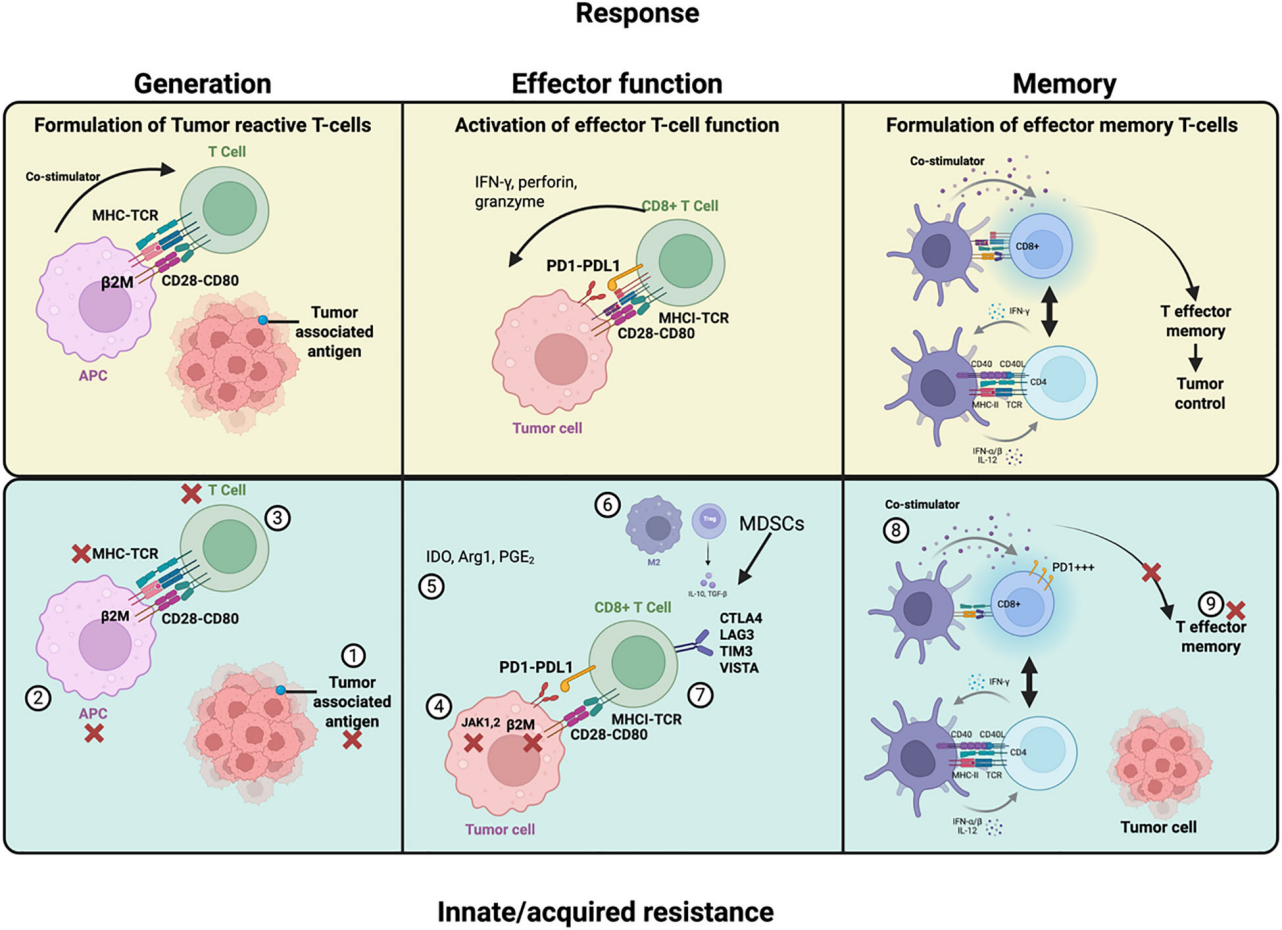
Upper panel: Illustrates the key stages in the development of tumor-specific T cells, their effector functions to eliminate cancer cells, and the generation of memory T cells for long-lasting immunity. Lower panel: Outlines the potential mechanisms driving innate or acquired resistance to ICI therapy, highlighting factors that impair effective anti-tumor immune responses and limit therapeutic success (1). Lack of sufficient or suitable neo-antigen (2) Impaired processing or presentation of tumor antigens (3) Impaired intratumoural immune infiltration (4) Impaired IFN-Gamma (5) Metabolic/inflammatory mediators (6) Immune suppressive cells (7) Alternative immune checkpoints (8) Severe T-cell exhaustion (9) T-cell epigenetic changes.

Tumor-intrinsic mechanisms encompass genetic, transcriptional, and epigenetic alterations that directly affect antigen presentation, interferon signaling, and oncogenic pathways within tumor cells. Acquired resistance usually results from tumor adaptation and immune escape that evolve under selective therapeutic pressure (25, 26). Recent clinical and genomic analyses in NSCLC have further characterized molecular features underlying acquired resistance to immune checkpoint inhibitors and identified recurrent alterations associated with late progression and immune evasion (27). Tumors characterized by a high load of non-synonymous mutations, such as melanoma, lung cancer, and bladder cancer, tend to exhibit some of the highest response rates to ICI therapy, particularly in NSCLC subtypes with smoking-associated mutational signatures and high tumor mutational burden (TMB) (28). Several large-scale analyses of clinical trials in NSCLC have confirmed that higher TMB is associated with improved outcomes to PD-1/PD-L1 blockade, independent of PD-L1 expression (29–32). For instance, some patients never respond to PD-1 or PD-L1 blockade despite high PD-L1 expression; these cases typically reflect primary resistance. Conversely, patients who relapse after an initial partial or complete response exhibit acquired resistance, often driven by neoantigen loss or JAK/STAT pathway mutations that disable immune recognition (33, 34). In NSCLC, loss-of-function JAK1 or JAK2 mutations have been identified in tumors refractory to PD-1 blockade, leading to impaired interferon-γ responsiveness and diminished CD8^+^ T-cell infiltration (35). Importantly, resistance to ICIs can also result from genetic alterations affecting components of the antigen processing and presentation machinery, such as major histocompatibility complex (MHC) class I molecules and β2M. Loss of β2M expression and downregulation of HLA class I have been reported in various tumors. Since β2M is essential for stable MHC class I surface expression, its loss impairs antigen presentation to cytotoxic T lymphocytes, thereby enabling immune evasion (36). Similar β_2_M deletions and HLA class I loss have been reported in NSCLC biopsies obtained after progression on anti-PD-1 therapy, correlating with reduced antigen presentation and poor clinical outcome (37). Consistent with these findings, NSCLC-specific analyses have demonstrated that impaired HLA class I antigen processing and presentation represent a mechanism of acquired resistance to PD-1 blockade in lung cancer (38). While clonal neoantigens are generally associated with favorable responses to ICI therapy, neoantigen evolution can contribute to acquired resistance through two distinct mechanisms: (a) the selective outgrowth of tumor cell clones that inherently lack neoantigen expression, allowing them to escape immune elimination; or (b) the emergence of genetic alterations that lead to the loss of neoantigen expression in previously immunogenic clones. Such dynamic changes in the tumor’s mutational landscape have been observed in patients who develop resistance following initial responsiveness to ICI treatment (39, 40). **Figure 2** illustrates the critical immunological events required for effective anti-tumor responses and the mechanisms, such as impaired neoantigen presentation and T-cell exhaustion, that drive innate and acquired resistance to ICIs in NSCLC. The recently described innate PD-1 resistance (IPRES) gene signature encompasses a collection of immunosuppressive cytokines, epithelial–mesenchymal transition (EMT) related transcription factors, and pro-angiogenic molecules, all of which have been linked to intrinsic resistance to PD-1 blockade (41). Notably, patients who do not respond to PD-1 blockade often exhibit gene expression profiles associated with wound-healing processes, epithelial–mesenchymal transition (EMT), and resistance to MAPK pathway inhibition (41, 42). Remarkably, the IPRES signature includes the receptor tyrosine kinase AXL, whose overexpression is linked to a reversible cellular state characterized by resistance to BRAF and MEK inhibitors (BRAFi/MEKi) and activation of the NF-κB signaling pathway (41, 43). It is intriguing to consider that the IPRES may represent a multigenic, reversible transcriptional program, which could potentially be modulated to alter an individual’s responsiveness to immune checkpoint inhibitor therapy. Conversely, studies profiling long-term responders to immunotherapy in advanced NSCLC have highlighted distinct molecular and immune signatures predictive of durable benefit (44).

#### 2.1.2 Tumor-extrinsic mechanisms

Approaches that induce immunogenic cell death, such as chemotherapy and radiotherapy, or that enhance antigen presentation through the activation of innate immunity and dendritic cell function (e.g., type I interferons, Toll-like receptor ligands, LIGHT, and oncolytic viruses) may facilitate the generation or display of immunogenic neoantigens. These strategies are particularly valuable in tumors characterized by a non-inflammatory, immune cell-poor TME, where baseline immune activation is limited (22, 45). Inhibiting immunosuppressive factors such as VEGF, IL-10, and TGF-β can enhance dendritic cell migration, maturation, and antigen-presenting capacity. By relieving these inhibitory signals, T-cell priming is improved, thereby creating a more favorable immunological environment that can synergize with ICIs to promote effective anti-tumor responses (22, 45). In cases where HLA-presented neoantigens are inadequate to activate cytotoxic T lymphocytes effectively, natural killer (NK) cells can serve as an alternative immune effector mechanism. Therapies that target NK cell checkpoints, such as anti-KIR antibodies, may enable NK cells to recognize and eliminate tumor cells independently of T cell-mediated responses, offering a complementary strategy to overcome immune evasion (46). Certain oncogenic signaling pathways influence the nature and degree of immune cell infiltration within tumors. For example, biallelic loss of PTEN, as observed in a patient with an isolated non-responding lesion despite near-complete response to PD-1 inhibition, has been linked to reduced T cell infiltration, elevated CCL2 and VEGF expression, and resistance to PD-1 therapy (47, 48). In NSCLC, PTEN loss has likewise been associated with an “immune-excluded” phenotype and primary resistance to PD-1 inhibitors (49). Alterations in β-catenin/WNT signaling have been shown to reduce CCL4 production, resulting in decreased infiltration of CD103^+^ dendritic cells and subsequently weakened anti-tumor immune responses (50, 51). Consistently, β-catenin activation in NSCLC models suppresses CCL4-dependent dendritic-cell recruitment, producing immune exclusion similar to that observed in melanoma (52). In NSCLC, the immunologic consequences of oncogenic alterations are strongly context-dependent. Specifically, loss of the tumor suppressor STK11/LKB1 in the presence of a KRAS mutation promotes IL-6–mediated neutrophil recruitment, limits CD8^+^ T-cell infiltration, and upregulates exhaustion markers such as PD-1, CTLA-4, and TIM-3, collectively generating an ‘immune-cold’ tumor microenvironment and conferring resistance to PD-1/PD-L1 blockade (53–55). Large-scale genomic profiling of NSCLC cohorts confirmed that KRAS/STK11 co-mutations define an immunologically “cold” subset with minimal PD-L1 expression and poor response to immune checkpoint inhibitors (56). Another extrinsic mechanism contributing to immune resistance involves tumor-associated macrophages (TAMs). Driven by cytokines such as TGF-β and IL-10, hypoxia-induced HIF1α, and metabolic factors, TAMs polarize toward an immunosuppressive M2-like phenotype. These macrophages secrete CCL2, VEGF, and arginase-1, which recruit additional suppressive myeloid cells and deplete nutrients essential for T-cell activation, thereby fostering immune escape and tumor progression in NSCLC. An additional extrinsic mechanism contributing to immune resistance involves TAMs (57). Numerous factors contribute to the M2 polarization of TAMs, including cytokines such as TGF-β and IL-10, chemokines like CXCL4 and CCL5, growth factors such as M-CSF and VEGF, as well as hypoxic conditions within the tumor microenvironment (58, 59). Malignant cells can further promote this polarization by releasing lactic acid and hypoxia-inducible factor 1-alpha (HIF1α), reinforcing the immunosuppressive state. In addition, tumor-derived signals such as TNF-α induce TAMs to secrete chemokines (CCL2, CCL8) that recruit CCR2^+^ monocytes, amplifying the pro-tumoral milieu and enhancing immune evasion in NSCLC (58, 59). This reorganization distinguishes intrinsic tumor alterations from extrinsic immunosuppressive mechanisms, providing a clearer conceptual framework for understanding both primary and acquired resistance before discussing pharmacologic interventions in Section 3. Although similar resistance mechanisms occur across multiple solid tumors, NSCLC displays unique resistance signatures driven by its mutational landscape, smoking-related carcinogens, and lung-specific immune microenvironment.

Tumor cells can escape T cell-mediated destruction by downregulating surface expression of major MHC molecules, particularly those of the MHC class I pathway, which is primarily responsible for presenting tumor antigens to cytotoxic T lymphocytes. As a result, defects in MHC class I antigen presentation are more commonly observed in resistant tumors compared to impairments in the MHC class II pathway. However, emerging evidence suggests that in NSCLC, altered expression of both MHC class I and II molecules has been correlated with poor PD-1/PD-L1 therapy response. A recent study (60) showed that MHC class II downregulation contributes to immune escape in resistant NSCLC tumors (61–64). The critical role of interferon-gamma (IFNγ) signaling in antitumor immunity is largely attributed to its ability to enhance MHC class I antigen presentation. This process depends on the coordinated expression of several key genes, including TAP1, TAP2, B2M, and immunoproteasome components such as PSMB8, PSMB9, and PSMB10. Tumor cells that lack responsiveness to IFNγ signaling often exhibit impaired or absent MHC class I antigen presentation, facilitating immune evasion. Supporting this, a 2001 study demonstrated that tumor cells deficient in IFNγ responsiveness could be rendered immunogenic through stable transfection of TAP1, leading to tumor rejection in immunocompetent (wild-type) mice, but not in T cell-deficient (Rag2−/−) animals, highlighting the T cell dependency of this immune response (65). Indeed, some tumor cells with deficient MHC class I expression rely on IFNγ pretreatment to restore effective antigen presentation. This cytokine stimulation is necessary to coordinate the expression of the peptide–MHC class I complex along with components of the antigen processing machinery, thereby enabling proper recognition by cytotoxic T lymphocytes (66).

Despite IFNγ signaling, flaws in the antigen processing system impair MHC class I expression on the cell surface (67). Such alterations may not only make tumors resistant to T cell-based immunotherapy but could also reflect immune-driven selection. In NSCLC patients, reduced β2M expression and HLA class I loss have similarly been observed following PD-1 blockade, correlating with disease progression (68, 69). Longitudinal biopsies from a distinct metastatic melanoma case demonstrated therapy-independent MHC class I deficiency resulting from β2M loss (70). An innovative computational method for HLA copy number estimation enabled evaluation of clonal and subclonal HLA loss of heterozygosity. The presence of multiple parallel, subclonal, and site-specific HLA LOH events, particularly in metastatic regions, indicates immune pressure on these tumors independent of therapy (71).

Individuals with microsatellite-unstable colorectal cancer, known for its high immunogenicity, exhibited comparable associations between immune pressure and genetic disruptions in antigen-processing components (36, 72). Additionally, β2M loss of heterozygosity correlated with reduced overall survival in two independent melanoma cohorts receiving immune checkpoint therapy (72). Additionally, genes that govern antigen presentation have been found. For instance, MEX3B, a post-transcriptional repressor of HLA-A, enables melanoma cells to evade tumor-specific T cells, as identified through an *in vitro* gain-of-function kinome screen. Notably, elevated MEX3B expression was observed in a subset of anti-PD1 non-responders (73). Interferon (IFN) signaling is essential for anti-tumor immunity by promoting antigen presentation, T cell infiltration, and the expression of effector molecules like PD-L1 and MHC class I. Type I (IFN-α/β) and type II (IFN-γ) IFNs primarily activate the JAK/STAT pathway. This mechanism underlies both immune surveillance and the therapeutic efficacy of immune checkpoint inhibitors. Upon receptor engagement, IFNs trigger JAK1 and JAK2 phosphorylation of STAT1 and STAT2, leading to their nuclear translocation and activation of immune-stimulatory genes, thereby supporting T cell recruitment, antigen presentation, and PD-L1 expression (74). However, loss-of-function mutations in JAK1, JAK2, or STAT proteins disrupt this signaling cascade, rendering cells unresponsive to interferon stimulation. Inactivating mutations in JAK1 or JAK2 have been identified in NSCLC tumors resistant to PD-1/PD-L1 therapy (75). These defects compromise T cell-mediated cytotoxicity, suppress interferon-stimulated gene activation, and diminish MHC class I expression, thereby limiting immune recognition of tumor cells and contributing to innate or acquired resistance to ICIs (74). Additionally, impaired interferon signaling prevents upregulation of chemokines like CXCL9 and CXCL10, which are essential for effector T cell recruitment to the tumor microenvironment. This leads to an “immune desert” phenotype, where cytotoxic cells fail to infiltrate the tumor despite ICI treatment (76, 77).

Disruption of the JAK/STAT pathway, a key regulator of anti-tumor immunity, poses a significant challenge to immunotherapy efficacy. Elucidating the molecular mechanisms underlying interferon resistance in NSCLC is essential for designing rational combination therapies such as cytokine treatments, STING agonists, or epigenetic modifiers to restore interferon sensitivity and resensitize tumors to ICIs (78, 79). In summary, tumor-intrinsic alterations such as loss of antigen presentation, defective interferon signaling, and oncogenic mutations (e.g., KRAS/LKB1, PTEN) collectively limit the clinical efficacy of PD-1/PD-L1 blockade in NSCLC, highlighting the necessity of integrating genetic profiling into immunotherapy design.

#### 2.1.3 Pharmacologic intervention

Monoclonal antibodies targeting co-inhibitory immune checkpoints, such as PD-1 and CTLA-4, have revolutionized the field of medical oncology owing to their demonstrated clinical efficacy across a broad spectrum of malignancies. These include melanoma, non-small cell lung cancer, renal cell carcinoma, bladder cancer, head and neck squamous cell carcinoma, microsatellite instability-high colorectal cancer, Merkel cell carcinoma, and Hodgkin lymphoma (80–82). Anti-PD-1 agents (nivolumab and pembrolizumab), anti-CTLA-4 therapy (ipilimumab), and combination regimens involving both checkpoints (nivolumab–ipilimumab) have become approved treatment options for melanoma, driven by the promising clinical outcomes of immune checkpoint inhibition. Long-term survival data indicate that 20% of melanoma patients treated with ipilimumab exhibit durable disease control or sustained responses five to ten years following the initiation of therapy (83). After three years of treatment with pembrolizumab (anti-PD-1), the response rate in melanoma patients was approximately 33%, with 70–80% of those initially responding maintaining a durable clinical benefit over time (84). Recent clinical findings indicate that patients with metastatic melanoma exhibit notably high response rates when treated with combination immunotherapy targeting both CTLA-4 and PD-1. This dual blockade strategy has achieved response rates approaching 58%, highlighting its potent anticancer potential. However, nearly half of the treated patients have experienced significant treatment-related toxicities, raising concerns about its safety profile (85). As such, robust long-term survival data are still urgently needed to fully validate the clinical benefit of this combined immunotherapeutic approach (86, 87).

Analysis of clinical trial data identifies three principal categories of patients undergoing immune checkpoint inhibitor therapy (1): those who exhibit an initial and sustained therapeutic response (responders) (2); those who show no clinical benefit from the outset (innate resistance); and (3) those who initially respond but later experience disease progression (acquired resistance) (21, 22, 45, 80). Distinguishing responders from non-responders remains a significant challenge in the context of ICI therapy, largely due to the diverse and often unpredictable patterns of clinical response. This heterogeneity can present within a single patient as stable disease with isolated progression, oligometastatic progression, or mixed responses across lesions. It may also be temporal, with initial disease stability followed by progression, or spatial, with differing responses across metastatic sites. Despite these complexities, the presence of a “tail” on survival curves reflects that a subset of patients can achieve durable disease control, potentially lifelong, following successful ICI treatment. This observation continues to fuel intensive research aimed at uncovering the underlying mechanisms of response and resistance, to extend durable benefit to a broader population of patients with advanced malignancies (88).

The mechanisms underlying both innate and acquired resistance to ICI therapy remain incompletely understood, largely due to the lack of comprehensive insights into the full spectrum of clinical, molecular, and immunological factors that correlate with therapeutic response and long-term benefit. Compounding this challenge is the limited availability of immune-competent preclinical models that accurately recapitulate the complexity of tumor-immune interactions observed in patients, and in which ICIs reliably induce tumor regression. This gap hampers efforts to systematically investigate resistance pathways and develop predictive biomarkers or effective combination strategies (89, 90). To properly contextualize the concepts of primary (inherent) and secondary (acquired) resistance to ICI therapy, it is essential to revisit the foundational “response” paradigm. This framework emphasizes a sequence of critical immunological events required for effective anti-tumor activity, each of which can be disrupted by the tumor itself or by components of the TME. Tumors may actively impede, bypass, or obstruct these phases through various mechanisms, while elements of the immune system and stromal cells within the TME may be co-opted to support immune evasion and promote resistance to ICI therapy. Understanding how these disruptions occur is vital for developing strategies to overcome resistance and enhance therapeutic efficacy (91, 92).

Tumor-intrinsic epigenetic alterations, commonly referred to as cell state modifications, are often driven by reversible chromatin modifications, such as the addition or removal of methyl or acetyl groups on DNA or histones. Epigenetic modifying agents (EMAs), including histone modifiers and DNA methyltransferase inhibitors, can influence tumor immunogenicity by modulating the expression of cancer-associated antigens (e.g., cancer-testis antigens), cytokines, and key components of the antigen processing and presentation machinery, such as TAP, HLA class I molecules, and β2M (93). Treatment with DNA methyltransferase (DNMT) inhibitors or EZH2 inhibitors has been shown to enhance responsiveness to immune checkpoint blockade and restore the production of Th1-type cytokines, thereby promoting a more effective anti-tumor immune response (94). Alterations in the methylation status of non-coding genomic regions can influence the efficacy of immunotherapy. Hypomethylating agents, such as 5-azacytidine, have been shown to modulate immunosuppressive cells within the tumor microenvironment, enhance innate immune signaling, affect T-cell priming and effector functions, and improve response to immune checkpoint inhibitors by activating endogenous retroviral elements (ERVs), thereby promoting a state of viral mimicry (95–97). Interestingly, tumor-specific endogenous retroviral elements (ERVs) have been associated with increased expression of immune-related genes and enrichment of cytolytic immune cells, suggesting a potential role in enhancing anti-tumor immune responses (98). Further investigation is needed to clarify the directionality and magnitude of the relationship between ERV expression and immune cell infiltration and activation. Nonetheless, there is increasing interest in leveraging ERV induction as a strategy to enhance responsiveness to PD-1 blockade. In this context, EMAs are being explored as potential adjuvants to immune checkpoint inhibitor therapy through their ability to modulate ERV activity (99).

Beyond their role in key oncogenic signaling cascades, long non-coding RNAs (lncRNAs) influence tumor immunology by facilitating immune evasion. According to Denaro et al., lncRNA-associated pathways modulate cancer immunity through various immune cells, including T cells, B cells, dendritic cells, macrophages, and myeloid cells. Several lncRNAs have been identified as both potential therapeutic targets and diagnostic biomarkers in cancer (100). Hence, exploring the therapeutic potential of lncRNAs involved in tumor-induced immunosuppression is of particular interest. To systematically summarize current findings, a literature search was conducted in the PubMed database using the terms “lncRNA or long noncoding RNA or long non-coding RNA, “ “immune suppression or immunosuppressive, “ and “tumor microenvironment.” This review includes studies addressing key immunological checkpoints (PD-L1, TIM-3, HLA-G) and immunosuppressive cell types (Tregs, MDSCs, TAMs) regulated by lncRNAs within the TME. Studies were excluded based on the following criteria: (a) those demonstrating lncRNA regulation of immune cells without cancer relevance; (b) those focusing on metastasis or progression without immune system involvement; and (c) those examining lncRNA roles in hematological malignancies (101). The reviewed literature indicates that lncRNA-mediated tumor immunosuppression can be categorized based on the cellular origin of the lncRNAs. Certain oncogenic lncRNAs modulate tumor immunogenicity within tumor cells by either suppressing tumor antigen production directly or enhancing the expression of immune checkpoints (e.g., PD-L1, IDO) and HLA-G indirectly. Additionally, lncRNAs secreted by tumor cells can be internalized by stromal cells recruited to the TME, which then release immunosuppressive factors contributing to tumor immune evasion. Most studies have concentrated on M2 macrophages, MDSCs, Tregs, and other immunoregulatory cells within the immunosuppressive TME. The lncRNAs implicated in tumor immune escape and their downstream targets are detailed in the following sections (101, 102).

Beyond genetic and signaling abnormalities, adaptive immune resistance mechanisms such as activation of alternative immune checkpoints and metabolic reprogramming within the TME also contribute to immune evasion in non-small cell lung cancer. These processes promote resistance to PD-1/PD-L1 and CTLA-4 blockade, thereby diminishing the effectiveness of existing ICIs (103).

A key adaptive mechanism involves the upregulation of non-redundant inhibitory receptors such as T cell immunoglobulin and mucin-domain containing-3 (TIM-3), LAG-3, and T cell immunoreceptor with Ig and ITIM domains (TIGIT) (104). These alternative immune checkpoints are frequently co-expressed with PD-1 on regulatory T cells and exhausted CD8^+^ T cells, especially under conditions of chronic antigen stimulation. Following ICI therapy, their expression commonly increases as a compensatory mechanism to sustain immune suppression (105, 106).

Dysfunctional T cells express TIM-3, which promotes immune tolerance by suppressing T cell proliferation and cytokine secretion. LAG-3, frequently acting in concert with PD-1, binds to MHC class II molecules to inhibit effector function by negatively regulating T cell activation and expansion (107). TIGIT suppresses T and NK cell activity by competing with the co-stimulatory receptor CD226 for interaction with CD155 and CD112 on antigen-presenting cells. In PD-1–resistant NSCLC, elevated expression of these receptors correlates with poor clinical outcomes. Therapeutic agents targeting TIM-3, LAG-3, and TIGIT are currently under clinical investigation to counteract immune exhaustion and reinvigorate T cell function, especially in combination with anti-PD-1/PD-L1 therapies. Metabolic reprogramming within the tumor microenvironment is another key resistance mechanism that promotes immune suppression and supports tumor development. Tumor cells actively alter their metabolic processes to accumulate metabolites that inhibit immune responses and reduce the availability of nutrients essential for immune effector cell activity. The adenosine-generating pathway and the indoleamine 2, 3-dioxygenase 1 (IDO1) pathway are two major metabolic routes associated with resistance to immune checkpoint inhibitors (108–112).

IDO1 catalyzes the breakdown of tryptophan into kynurenine, leading to local tryptophan depletion and the accumulation of metabolites that suppress T cell proliferation and facilitate regulatory T cell expansion. Elevated IDO1 expression in NSCLC is associated with an immunosuppressive microenvironment and has been linked to resistance to immune checkpoint blockade. Although early trials combining IDO1 and PD-1 inhibitors showed promise, subsequent clinical studies have yielded inconsistent outcomes, highlighting the complexity of targeting this pathway. Similarly, the adenosine signaling axis exerts strong immunosuppressive effects within the TME. Under hypoxic conditions, tumor cells activate ectonucleotidases CD39 and CD73, which convert extracellular ATP into adenosine. Accumulation of adenosine suppresses effector T cell function and cytokine release through A2A receptor (A2AR) activation. High adenosine levels are associated with poor immune infiltration and resistance to ICIs in lung cancer. As a result, inhibitors of CD73 and A2AR are currently under clinical investigation as potential combinatory agents in immunotherapy (113–115). The concurrent activation of metabolic reprogramming and alternative immune checkpoints in NSCLC poses major, yet targetable, obstacles to durable immunotherapy responses. Continued investigation into these mechanisms holds promise for overcoming resistance and extending the therapeutic benefits of ICIs to a wider patient population (**Table 1**) (116, 117). Overall, these tumor-extrinsic factors, including immunosuppressive cell infiltration, metabolic reprogramming, and lncRNA-mediated regulation, define the non-cell-autonomous landscape of immune resistance in NSCLC and represent actionable targets for combinatorial immunotherapy.

**Table 1:** Summary of tumor-intrinsic and extrinsic resistance mechanisms to ICIs, with their functions, pros, cons, and key references.

Mechanism	Type	Description	Mechanistic impact/effect on immunity	Clinical consequences/therapeutic implications	References
MHC Class I Downregulation	Tumor-Intrinsic	CD8+ T lymphocytes are less likely to present antigens when HLA or β2-microglobulin (B2M) genes are mutated or lost.	CD8+ T lymphocytes are less likely to present antigens when HLA or β2-microglobulin (B2M) genes are mutated or lost.	Causes tumor escape and a poor ICI response.	(118, 119)
Defective Antigen Processing Machinery (APM)	Tumor-Intrinsic	TAP1, TAP2, and LMP7 mutations impair appropriate peptide presentation by MHC I.	Reduces the ability to recognize immune	Decreases the effectiveness of ICIs and impairs the cytotoxic T cell response.	(120, 121)
Defective IFN-γ Signaling (JAK1/2, STATs)	Tumor-Intrinsic	Mutations that cause loss of function affect antigen presentation and immunological activation.	reduces inflammation and immunological pressure	Promotes resistance to the inhibition of PD-1/PD-L1.	(122)
PTEN Loss	Tumor-Intrinsic	T cell infiltration is decreased when tumor suppressor PTEN is lost because it increases PI3K-AKT signaling.	Increases immune evasion and tumor growth.	linked to elevated VEGF expression and a poor response to ICI	(123)
β-catenin/WNT Pathway Activation	Tumor-Intrinsic	inhibits CCL4, which lowers T cell priming and dendritic cell recruitment.	prevents inflammation and immune detection	Produces a modest ICI response and non-T cell-inflamed TME.	(124, 125)
STK11/LKB1 Loss	Tumor-Intrinsic	causes T cell fatigue and immunosuppressive neutrophil recruitment.	encourages the growth of tumors	Low levels of PD-L1 expression and immunotherapy resistance	(56)
IPRES (Innate PD-1 Resistance) Signature	Tumor-Intrinsic	Gene enrichment associated with angiogenesis, inflammation, and EMT	Provides widespread resistance to treatment.	needs combinatorial techniques to get beyond	(35)
Epigenetic Modifications	Tumor-Intrinsic	Immune genes and antigen presentation are silenced by aberrant methylation and histone modification.	Reversible; epigenetic medications can target	For maximum impact, ICIs must be combined.	(126)
Alternative Immune Checkpoints (TIM-3, LAG-3, TIGIT)	Tumor-Extrinsic	increased during PD-1 inhibition, sustaining T cell fatigue	Possibility of using combinatorial immunotherapy	Variability in patient reaction and redundancy	(127, 128)
lncRNA-Mediated Immunosuppression	Tumor-Extrinsic	Long non-coding RNAs influence cytokines, Treg recruitment, and immunological checkpoints.	Novel target and biomarker classes	Complex regulation that is yet not fully understood	(129)
IDO1 Pathway Activation	Tumor-Extrinsic	reduces effector T cells, raises kynurenine, and depletes tryptophan.	Targetable with inhibitors of IDO1	Clinical trial outcomes that vary; compensating pathways	(130, 131)
Adenosine Pathway (CD39/CD73)	Tumor-Extrinsic	T and NK cells are suppressed when extracellular ATP is broken down to adenosine.	Tumor survival is enhanced by an immunosuppressive niche.	Combination treatments may be necessary for inhibition.	(132, 133)
Low Tumor Mutational Burden (TMB)	Tumor-Intrinsic	Reduced neoantigen burden due to fewer mutations lowers immunological visibility.	Prevents immunological recognition and destruction	forecasts a subpar reaction to ICIs	(134, 135)

## 3 Pharmacological strategies to overcome resistance

Immune resistance to ICIs in NSCLC manifests through distinct patterns that influence therapeutic design. Primary resistance reflects an inherent lack of response to ICIs, typically due to absent T-cell infiltration or low neoantigen burden, requiring combination or priming strategies. Acquired resistance develops after an initial response, driven by secondary immune escape mechanisms such as MHC downregulation or neoantigen loss, necessitating sequential or re-sensitization approaches. Adaptive resistance represents a dynamic feedback process, where immune activation itself induces compensatory inhibitory pathways (e.g., PD-L1, TGF-β), which can often be reversed through targeted or metabolic interventions (**Table 2**).

**Table 2:** Summary of emerging pharmacological strategies to overcome immune checkpoint inhibitor resistance in NSCLC.

Category	Mechanism/target pathway	Representative agents/approaches	Mechanistic basis	Clinical status/key trials	Current insight	Reference
1. Alternative Immune Checkpoint Blockade	Inhibition of compensatory receptors (LAG-3, TIGIT, TIM-3, VISTA)	e.g., Relatlimab (anti LAG-3), Tiragolumab (anti TIGIT)	Restore effector T-cell function by blocking redundant inhibitory signals	Relatlimab + nivolumab: phase 2/3 (RELATIVITY-047) showed benefit. Tiragolumab + atezolizumab in NSCLC (CITYSCAPE/SKYSCRAPER) with mixed results.	Promising immune reactivation, but mixed efficacy in NSCLC; biomarker refinement ongoing	(136, 137)
2. Tumor Microenvironment (TME) Modulation	Reprogramming immunosuppressive macrophages & cytokine milieu (e.g., CSF1R, TGF-β, IDO1, Arginase)	CSF1R inhibitors; TGF-β inhibitors; IDO1/Arginase inhibitors	Deplete TAMs/MDSCs, restore T-cell infiltration, block suppressive cytokines	Preclinical and early phase data show potential.	Converts “cold” to “hot” tumors; early synergy with ICIs observed but translation in NSCLC still early	(138, 139)
3. Oncogenic Pathway Co-Targeting	EGFR, KRAS(G12C), MAPK signalling blockade alongside immunotherapy	e.g., EGFR/ALK TKIs, KRAS G12C inhibitors + ICIs	Reduce oncogene-driven immune exclusion; enhance antigenicity	Review of resistance mechanisms highlights oncogenic signalling as intrinsic resistance factor. Trials ongoing in specific genotypes	Effective in defined genotypes; toxicity and timing/co-sequence remain limiting	
4. Epigenetic Reprogramming	DNMT/HDAC inhibition to restore antigen processing and T-cell infiltration	DNMT inhibitors (e.g., azacitidine), HDAC inhibitors (e.g., entinostat, vorinostat)	Reactivate silenced immune genes, enhance MHC-I expression & cytokine signalling	Preclinical and early clinical studies in NSCLC and other tumour types show priming effect.	Reverses immune exhaustion; combination strategies being refined	(140)
5. Cytokine-Based Therapies	Engineered cytokines (IL-2 variants, IL-15, IL-7) to amplify T/NK cell activation	Modified IL-2 (bempegaldesleukin), IL-15 agonists	Amplify CD8^+^ and NK activation while minimising Treg expansion	Under clinical evaluation; mechanistic basis supported by reviews.	Enhanced T-cell activity; phase III results so far mixed; side-effect profiles important	(141)
6. Adoptive Cell & Vaccine Therapies	Neoantigen-based vaccines; TIL transfer; CAR-T/TCR-T in solid tumours	Neo-antigen vaccines (e.g., NEO-PV-01), TIL/TCR-T therapies	Reintroduce tumour-reactive T cells; expand neoantigen immunity	Early phase trials ongoing in NSCLC and other solid tumours.	Personalized, durable immunity possible; logistical and cost challenges remain	(142)
7. Metabolic Reprogramming	Targeting adenosine, arginine, glutamine metabolism in TME	A2AR antagonists (e.g., ciforadenant), arginase inhibitors, glutaminase inhibitors (e.g., CB-839)	Reverse nutrient depletion and metabolite-driven immunosuppression	Strong mechanistic rationale; early phase trials underway.	Restores metabolic fitness of immune cells; still early in clinical translation	(143)
8. Combination and Sequential Strategies	Rational integration of ICIs with above agents (dual/triple therapy)	e.g., PD-1 + LAG-3 + TGF-β blockade; epigenetic + ICI; metabolic + ICI	Overcome multifactorial resistance by multi-pathway targeting	Multiple ongoing phase I-III studies.	Represents next-generation precision immuno-oncology paradigm; toxicity and optimal sequencing remain key	(140)

### 3.1 Targeting alternative immune checkpoints

Resistance to PD-1/PD-L1 blockade in NSCLC has highlighted the need to target compensatory inhibitory receptors that mediate T-cell exhaustion and immune evasion. Key non-redundant checkpoints, including LAG-3, TIGIT, TIM-3, and VISTA, are frequently upregulated within the TME of resistant tumors, offering mechanistically distinct avenues for therapeutic intervention.

**Figure 3:**
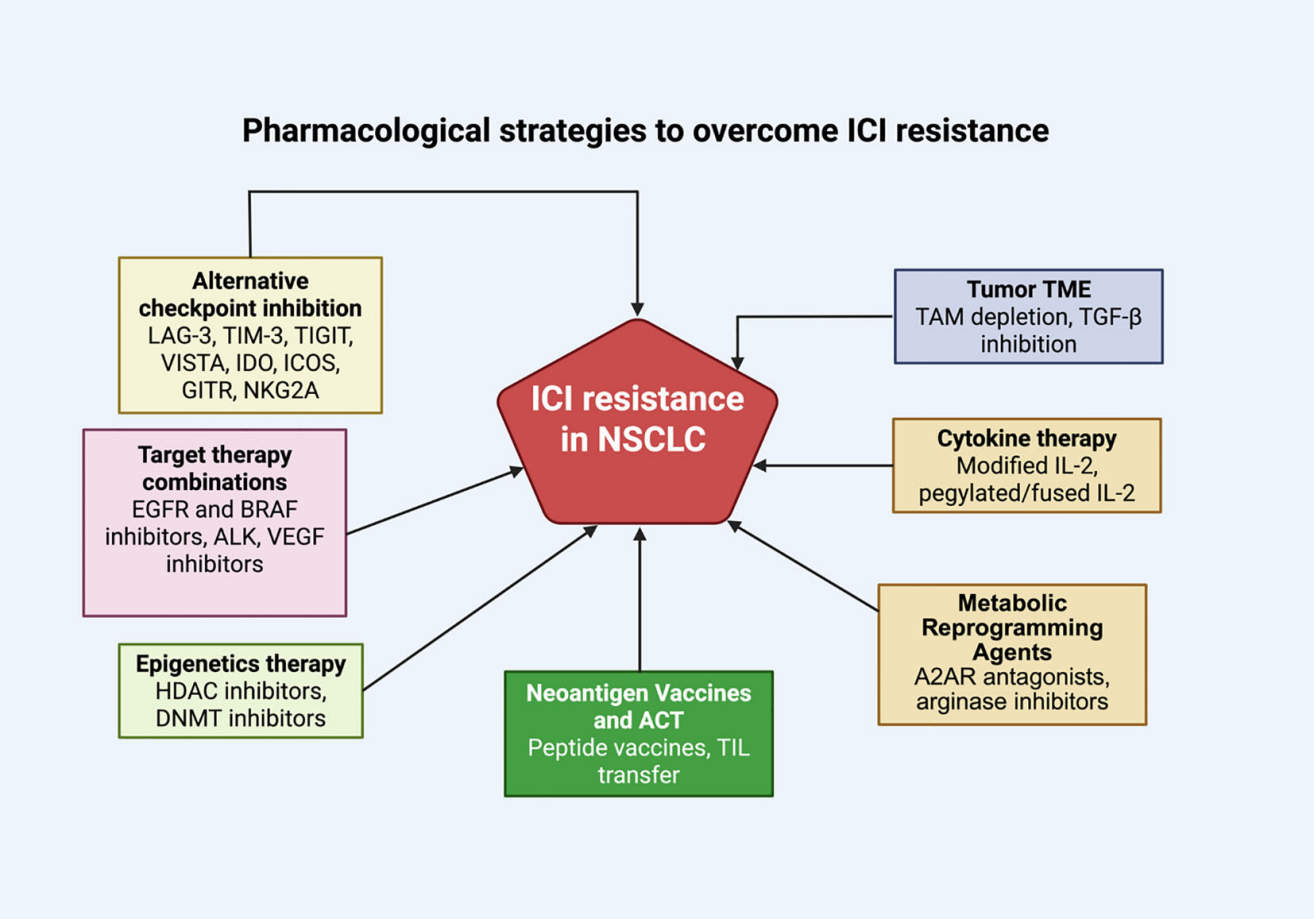
Pharmacological Strategies to Overcome Immune Checkpoint Inhibitor (ICI) Resistance in Non-Small Cell Lung Cancer (NSCLC). A schematic overview of therapeutic strategies aimed at overcoming ICI resistance in NSCLC. These approaches include alternative immune checkpoint blockade (e.g., LAG-3, TIM-3, TIGIT, VISTA), targeted therapy combinations (e.g., EGFR, BRAF, ALK, VEGF inhibitors) (141), modulation of the tumor microenvironment (e.g., TAM depletion, TGF-β inhibition), cytokine-based therapies (e.g., modified IL-2 formulations), epigenetic modulators (e.g., HDAC and DNMT inhibitors), metabolic reprogramming agents (e.g., A2AR antagonists, arginase inhibitors), and neoantigen-based vaccines or adoptive cell transfer (ACT). These combinatorial or standalone strategies are being explored to restore anti-tumor immunity and enhance responsiveness to checkpoint blockade therapy.

Among these receptors, LAG-3 and TIGIT have shown the most clinical progress. Dual PD-1 and LAG-3 blockade has demonstrated promising activity in early-phase NSCLC trials, suggesting that this combination can restore T-cell effector functions and re-sensitize resistant tumors. Similarly, TIGIT inhibition has shown synergistic effects with PD-L1 blockade, improving antitumor activity, particularly in tumors with high PD-L1 expression. TIM-3 and VISTA remain emerging targets, currently under investigation for their potential to reverse terminal T-cell exhaustion and macrophage-driven immune suppression. Relatlimab, a monoclonal antibody targeting LAG-3, has shown clinical promise. Early data from the RELATIVITY-073 trial (NCT04623775) indicate potential benefit in PD-1–resistant NSCLC as well. Ongoing clinical trials are now exploring its efficacy in non-small cell lung cancer. In NSCLC, preliminary data suggest that dual LAG-3 and PD-1 blockade can re-sensitize resistant tumors to immune activation, supporting ongoing phase II–III evaluation (145–147). TIGIT (T cell immunoreceptor with Ig and ITIM domains) is another inhibitory receptor co-expressed with PD-1 on tumor-infiltrating lymphocytes. It suppresses T and NK cell activation by competing with the co-stimulatory receptor CD226 for binding to the shared ligands CD112 and CD155 on antigen-presenting cells (148–150). TIGIT expression is associated with poorer prognosis and diminished response to ICIs in NSCLC. Tiragolumab, a monoclonal antibody targeting TIGIT, has demonstrated initial clinical benefit when combined with atezolizumab (151, 152), especially in tumors with high PD-L1 expression. Ongoing Phase III trials are assessing the effectiveness of this combination in broader NSCLC patient populations (153, 154). Recent updates from the SKYSCRAPER-01 and SKYSCRAPER-02 phase III trials have shown that tiragolumab plus atezolizumab did not meet their primary endpoints for overall or progression-free survival, leading to the discontinuation of several tiragolumab studies within the clinical development program (155, 156). Furthermore, Rilvegostomig, a novel bispecific PD-1 × TIGIT antibody, has demonstrated early clinical activity and favorable safety in advanced NSCLC, according to interim results from the ARTEMIDE-Lung01 and ARTEMIDE-Lung02 trials (155, 156). **Figure 3** illustrates a range of pharmacological strategies, such as alternative checkpoint inhibitors and TME modulation, designed to counteract ICI resistance and enhance anti-tumor immunity in NSCLC.

TIM-3 (T cell immunoglobulin and mucin-domain containing-3) is another key inhibitory receptor linked to terminal T cell exhaustion. It is co-expressed with PD-1 on dysfunctional T cells and interacts with ligands such as galectin-9 and phosphatidylserine (157, 158). Preclinical evidence indicates that blocking TIM-3 can potentiate antitumor immunity and restore exhausted T cell function, particularly when combined with PD-1 inhibitors. Several anti-TIM-3 antibodies, including sabatolimab, are currently under early-phase investigation for the treatment of solid tumors, including non-small cell lung cancer (159). VISTA (V-domain Ig suppressor of T cell activation) is a novel immune checkpoint predominantly expressed by tumor-infiltrating macrophages and myeloid-derived suppressor cells (160). VISTA contributes to maintaining an immunosuppressive environment and may become upregulated after PD-1/PD-L1 blockade. Although still in preclinical and early clinical stages, VISTA antagonists hold potential to enhance T cell activation and counteract immune resistance in refractory tumors (161). Collectively, targeting these alternative immune checkpoints presents a rational, mechanism-driven strategy to reinvigorate antitumor immunity in NSCLC patients unresponsive to standard immune checkpoint inhibitors. Ongoing research aims to determine whether combinatorial approaches can effectively translate preclinical synergy into durable clinical outcomes. Together, these findings underscore that targeting alternative inhibitory checkpoints such as LAG-3, TIGIT, TIM-3, and VISTA can reinvigorate antitumor immunity in NSCLC, supporting a paradigm shift toward multi-checkpoint blockade strategies.

### 3.2 Modulating the tumor microenvironment

**Figure 4:**
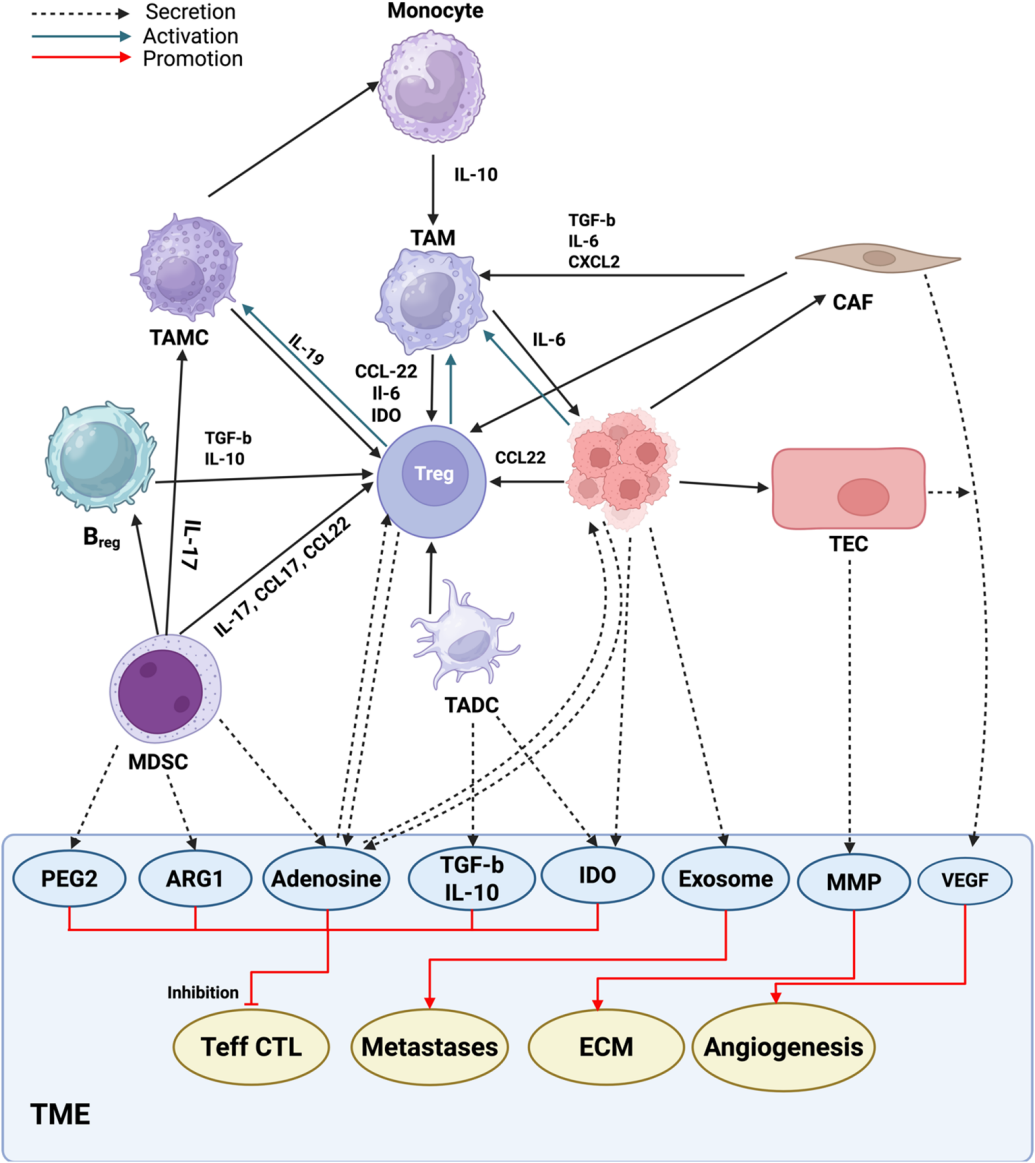
Within the tumor microenvironment (TME), extensive cross-talk occurs between immune and non-immune cells through the secretion of various soluble factors and direct interactions. Key molecules involved include prostaglandin E2 (PGE2), arginase-1 (ARG1), adenosine, transforming growth factor β (TGF-β), interleukin-10 (IL-10), indoleamine 2, 3-dioxygenase (IDO), exosomes, matrix metalloproteinases (MMPs), and vascular endothelial growth factor (VEGF), among others. These mediators collectively suppress the function and proliferation of CD4^+^ and CD8^+^ T cells while promoting angiogenesis, extracellular matrix remodeling, and tumor metastasis.

This section focuses on tumor-extrinsic mechanisms of immune resistance—factors arising from the TME that suppress immune activation through cellular and cytokine-mediated pathways (162–168). Immunologically ‘cold’ tumors evade immune detection through defective antigen presentation, immune exhaustion, and the establishment of a suppressive tumor microenvironment that limits T-cell infiltration. In NSCLC, the tumor microenvironment plays a pivotal role in shaping response and resistance to immune checkpoint inhibitors (162–168). Clinical studies have shown that TAMs contribute to immunosuppression in cancer (169, 170). In most cancers, TAMs exhibit a phenotype resembling M2 polarization characterized by immunosuppressive functions and the promotion of tumor growth and metastasis (171). For example, through CSF-1R signaling, TAM-derived CCL8 enhances tumor cell secretion of colony-stimulating factor 1 (CSF-1), a key factor required for the survival and differentiation of macrophages and dendritic cells (172, 173). Therapeutically, inhibition of CSF-1R signaling using small-molecule or monoclonal antibodies (such as pexidartinib or emactuzumab) has been shown to deplete or reprogram TAMs, thereby enhancing cytotoxic T-cell infiltration and improving responses to PD-1/PD-L1 blockade in NSCLC. A major consequence of TAM accumulation is the suppression of T cell effector functions. For instance, TGF-β signaling skews CD4^+^ T cell differentiation toward immunosuppressive Th2 and Treg lineages, thereby dampening antitumor immune responses (174). Targeting TGF-β with selective inhibitors or neutralizing antibodies has demonstrated the ability to reverse macrophage-induced immunosuppression, restore CD8^+^ T-cell activity, and synergize with checkpoint blockade in preclinical and early clinical studies of NSCLC. Furthermore, TGF-β signaling impairs dendritic cell (DC) infiltration into tumor sites and suppresses the effector functions of both CD8^+^ T cells and NK cells, further contributing to immune evasion within the tumor microenvironment (175). T cells responding to TAM-secreted stimuli exhibit increased expression of immunosuppressive immune checkpoint markers in the TME, including PD-1, CTLA-4, Lag3, and TIM3 (176). TAMs frequently express higher levels of PD-L1 than tumor cells, and PD-L1 signaling within TAMs can directly impair their tumor cell phagocytic capacity, further weakening antitumor immunity (177). TAMs contribute to metabolic remodeling of the tumor microenvironment by generating enzymes that interfere with T cell signaling or by depleting essential amino acids required for T cell survival and expansion. For instance, TAM-derived arginase 1 (Arg-1) depletes L-arginine, leading to downregulation of the T cell receptor (TCR) ζ chain and resulting in impaired function of tumor-infiltrating lymphocytes (178–180). Another example is the excessive production of IDO by TAMs and tumor cells, which depletes tryptophan in the tumor microenvironment, an essential amino acid for T cell survival, thereby contributing to immune suppression (181). To counteract tryptophan depletion, IDO1 inhibitors (such as epacadostat and linrodostat) are being evaluated in combination with PD-1 blockade to restore T-cell metabolic fitness and enhance antitumor efficacy in NSCLC. **Figure 4** highlights the intricate interactions within the tumor microenvironment, driven by immunosuppressive mediators like arginase-1 and adenosine, which contribute to immune suppression and tumor progression in NSCLC. Representative pharmacologic interventions targeting TAMs and the immunosuppressive TME, including CSF1R blockade, TGF-β inhibition, and IDO1 pathway modulation, are summarized in **Table 3**.

**Table 3:** summarizes current pharmacological strategies to overcome key mechanisms of ICI resistance in NSCLC, classified by their target pathways and modes of action.

Resistance mechanism	Therapeutic strategy	Representative agents	Clinical trial/phase	Mode of action	Reference
Loss of Antigen Presentation	Epigenetic Therapy	Entinostat, Azacitidine	NCT03854474 (Phase II); NCT01928576 (Phase II)	Upregulate MHC-I expression, restore antigen processing	(144)
T-cell Exhaustion	Cytokine Modulation/Epigenetic Reprogramming	IL-7, IL-15, HDAC inhibitors	NCT04332653 (Phase I/II); NCT04250156 (Phase I)	Reinvigorate exhausted T cells, enhance effector function, and memory formation	
Alternative Checkpoint Upregulation (LAG-3, TIGIT, TIM-3)	Checkpoint Inhibitor Combinations	Relatlimab (LAG-3), Tiragolumab (TIGIT), Anti-TIM-3	SKYSCRAPER-01 (Phase III) – did not meet primary endpoint; ARTEMIDE-01 (Phase I) – favorable safety/efficacy	Block redundant inhibitory pathways on T cells to restore anti-tumor immunity	
Immunosuppressive TME	TME Modulation	Anti-CSF1R, TGF-β inhibitors, IDO1 inhibitors	NCT03927157 (Phase II); NCT03717070 (Phase I/II)	Deplete TAMs/MDSCs, inhibit suppressive cytokines, and metabolic enzymes	
Oncogenic Pathway Activation	Targeted Therapy Combinations	EGFR-TKIs, KRAS inhibitors (Sotorasib), MEK inhibitors	NCT04135395 (Phase I/II); NCT04613596 (Phase II)	Reduce oncogene-driven immune evasion and resistance	
Cytokine Dysregulation	Cytokine Therapy	Bempegaldesleukin (NKTR-214), Modified IL-2	NCT03635983 (Phase II); NCT04494353 (Phase I)	Enhance T-cell proliferation and cytotoxicity in TME	
Lack of Tumor Immunogenicity	Neoantigen-Based Vaccines/ACT	NEO-PV-01, TILs, TCR-T, CAR-T	NCT03639714 (Phase I); NCT04614103 (Phase II)	Introduce or amplify tumor-specific T-cell responses	
Adenosine and Metabolite Accumulation	Metabolic Reprogramming Agents	Ciforadenant (A2AR antagonist), Arginase inhibitors	NCT04262856 (Phase II)	Reverse immunosuppressive metabolic conditions in TME	

### 3.3 Combining ICIs with oncogenic pathway inhibitors

In contrast to TME-driven extrinsic mechanisms, tumor-intrinsic pathways defined by oncogenic signaling and intracellular genomic alterations also drive immune evasion and resistance to ICIs. Oncogenic driver mutations actively reshape the tumor immune landscape and contribute to tumorigenesis, often reinforcing resistance to immune checkpoint blockade (182, 183). Combining ICIs with targeted therapies aimed at specific oncogenic pathways presents a potential strategy to restore tumor sensitivity to immunotherapy. This approach is under active investigation, particularly in NSCLC subsets harboring mutations in EGFR, KRAS(G12C), or components of the MAPK pathway (182, 183). EGFR-mutant NSCLC poses a notable challenge in immuno-oncology; despite occasional PD-L1 expression, these tumors typically exhibit low TMB, limited CD8^+^ T cell infiltration, and an overall immunologically “cold” tumor microenvironment (184). These characteristics have contributed to poor clinical responses to PD-1/PD-L1 inhibitors. Although combining EGFR tyrosine kinase inhibitors (TKIs), such as osimertinib, with ICIs appears theoretically sound, early-phase trials have highlighted concerns about elevated toxicity, most notably, a high incidence of immune-related pneumonitis. As a result, ongoing research is focused on developing carefully timed treatment sequences or biomarker-driven combination strategies to minimize risk and enhance therapeutic efficacy (185–187). KRAS(G12C) mutations, present in approximately 13% of non-small cell lung cancer cases, represent a therapeutic target closely linked to immunotherapy resistance. Although KRAS-mutant tumors generally exhibit elevated TMB and PD-L1 expression, making them initially more responsive to ICI, this responsiveness is often diminished by co-occurring mutations in genes such as KEAP1 or STK11. Sotorasib, a KRAS(G12C) inhibitor, has demonstrated clinical activity as a monotherapy, and ongoing trials are evaluating its combination with PD-1 inhibitors. The therapeutic aim is to reprogram tumor signaling to enhance immune cell infiltration and suppress immunosuppressive cytokine production. Early evidence indicates that combining ICIs with KRAS(G12C) inhibitors may potentiate immune activation, particularly in the absence of additional immunosuppressive genomic alterations (188–192). Recent analyses have further evaluated whether intensified dual immune-checkpoint blockade can overcome the immunoresistant phenotype conferred by STK11 and KEAP1 co-mutations. *Post-hoc* data from CheckMate 227 and CheckMate 9LA trials indicated that ipilimumab plus nivolumab achieved numerically improved survival and durable responses in KRAS-mutant NSCLC with concurrent STK11 or KEAP1 loss compared with PD-1 monotherapy (193). Similarly, subgroup analyses of the POSEIDON and MYSTIC studies demonstrated that durvalumab plus tremelimumab partially restored immune activation and clinical benefit in these genomically defined subsets (194). These findings suggest that dual-checkpoint inhibition may mitigate the “immune-cold” microenvironment characteristic of STK11/KEAP1-mutant NSCLC and warrant continued investigation in prospective biomarker-stratified trials (195).

Research is also exploring the potential of MEK inhibitors to reprogram the tumor immune microenvironment. As downstream effectors of KRAS and other receptor tyrosine kinases, MEK inhibitors target chronic MAPK signaling a pathway linked to impaired antigen presentation and T cell exclusion. In preclinical models, MEK inhibition has been shown to promote T cell infiltration, diminish immunosuppressive myeloid cell populations, and upregulate MHC class I expression. When combined with PD-1/PD-L1 blockade, MEK inhibitors may help overcome adaptive resistance and reinstate antitumor immunity. However, optimal timing and dosing are critical, as excessive MAPK inhibition can dampen T cell activation (196, 197). In summary, combining immune checkpoint inhibitors with targeted therapies directed at oncogenic pathways represents a precision-based strategy to counteract intrinsic immune resistance in NSCLC. Tailoring these combinations to specific molecular subtypes necessitates thoughtful integration of treatment timing, toxicity profiles, and the immunological landscape. Ongoing clinical trials will be instrumental in refining these approaches and determining which patient populations are most likely to derive clinical benefit.

### 3.4 Epigenetic modulators

Additionally, epigenetic remodeling plays a crucial role in T cell activation, differentiation, effector function, and exhaustion, primarily through mechanisms such as DNA methylation and histone deacetylase (HDAC) regulation (198–200). Preclinical studies indicate that treatment of NSCLC cells with DNMT and HDAC inhibitors enhances interferon-α/β signaling, upregulates components of the antigen presentation machinery, and improves tumor control. These effects are linked to increased T cell infiltration within the tumor microenvironment and the reversal of T cell exhaustion (201).

However, demethylation can also amplify inhibitory signaling pathways that suppress T cell function, as evidenced by the upregulation of PD-L1 expression on NSCLC cells following *in vitro* treatment with azacitidine (202). Comparable effects have been observed in NSCLC models, where HDAC inhibitors increased PD-L1 expression and restored interferon pathway activity (203).

Numerous clinical trials have explored combining ICIs with epigenetic therapies in NSCLC to overcome resistance and enhance immunotherapy efficacy. In the ENCORE 601 trial (NCT02437136), patients who had progressed on prior ICIs were randomized to receive nivolumab with the HDAC inhibitor entinostat. This study revealed immunologic reprogramming and clinical benefit in a subset of patients, suggesting that epigenetic modulation can re-sensitize resistant tumors. Similarly, a trial (NCT01928576) evaluated the DNA methyltransferase (DNMT) inhibitor azacitidine combined with either nivolumab or ipilimumab to enhance antigen presentation and immune activation. Another early-phase trial (NCT02959437) assessed durvalumab (anti-PD-L1) with azacitidine in advanced solid tumors, including NSCLC, showing manageable safety and potential immunostimulatory effects. In another study (NCT02638090), the HDAC inhibitor vorinostat combined with pembrolizumab showed partial reversal of immune suppression in patients with advanced NSCLC. Additionally, decitabine, a DNMT inhibitor, has been studied in combination with nivolumab (NCT03250273) for its capacity to demethylate immune-related genes and restore checkpoint inhibitor responsiveness. Collectively, these trials underscore the therapeutic promise of epigenetic agents in enhancing ICI responses and remodeling the tumor microenvironment, particularly in immunologically “cold” or treatment-refractory NSCLC subtypes. Collectively, these data suggest that epigenetic reprogramming can convert immunologically ‘cold’ NSCLC tumors into ‘hot’ phenotypes, restoring responsiveness to immune checkpoint blockade when used in rational combination regimens.

### 3.5 Cytokine-based strategies

Since the discovery of interleukin-2 (IL-2) in 1976 as a powerful T cell growth factor, it has attracted considerable interest as a potential cancer therapy. However, IL-2 plays a dual and context-dependent role in immune regulation, capable of both enhancing and suppressing immune responses through its interaction with various immune cell subsets. Effective therapeutic use of IL-2 requires selectively amplifying anti-tumor immune activation while limiting the expansion of immunosuppressive cell populations and associated toxicities. Innovative approaches such as fusion proteins and pegylated IL-2 constructs have been developed to optimize its antitumor function and broaden its therapeutic window.

First discovered in the supernatant of human peripheral blood leukocytes activated by phytohemagglutinin, the 15.5-kDa cytokine IL-2 plays a central role in T-cell proliferation and immune activation (204, 205). In addition to sustaining CD4^+^ T cell populations, IL-2 drives clonal expansion of T cells and supports the differentiation of naïve CD8^+^ T cells into effector memory and terminal effector phenotypes. Beyond its established role in T cell proliferation, IL-2 also enhances the cytotoxic activity of lymphokine-activated killer cells and NK cells (206). Exposure to IL-2 enhances the production of granzyme B, perforin, and pro-inflammatory cytokines by activated T and NK cells. IL-2 is also critical for the generation of T helper 9 (Th9) cells, primes differentiation toward Th1 and Th2 lineages, and suppresses the development of Th17 and T follicular helper (TFH) cells. However, IL-2 simultaneously exerts immunosuppressive effects by supporting the expansion and stability of regulatory T cells, particularly the CD4^+^CD25^+^Foxp3^+^ Treg subset (207).

IL-2 engagement with its receptor (IL-2R) triggers the activation of major intracellular signaling cascades, including the JAK/STAT, PI3K/AKT, and MAPK pathways (208). The interleukin-2 receptor (IL-2R) is composed of three subunits: α (CD25), β (CD122), and γ (CD132). IL-2 binds with low affinity (Kd \~10^−8^ M) to IL-2Rα alone, with intermediate affinity (Kd \~10^−9^ M) to the IL-2Rβ/γ dimer, and with high affinity (Kd \~10^−11^ M) to the complete heterotrimeric receptor composed of all three subunits. L-2Rα is predominantly expressed by mature dendritic cells, CD56high NK cells, B cells, Tregs, activated CD4^+^ and CD8^+^ T cells, and endothelial cells. In contrast, IL-2Rβ and IL-2Rγ are mainly expressed by monocytes, NK cells, neutrophils, memory CD8^+^ T cells, and Tregs (207–209). CD8^+^ T cells and NK cells predominantly express the intermediate-affinity dimeric IL-2 receptor composed of IL-2Rβ and IL-2Rγ subunits (210). In contrast, Tregs primarily express the high-affinity trimeric IL-2 receptor, which requires the co-expression of all three subunits IL-2Rα, IL-2Rβ, and IL-2Rγ for optimal IL-2 binding (208).

### 3.6 Adoptive cell therapies and vaccines

Neoantigens are central to initiating a potent tumor-specific immune response. Tumors with high mutational burden are believed to possess an enhanced ability to stimulate CD4^+^ and CD8^+^ T cell responses via the presentation of immunogenic neoantigens. These neoepitopes tumor-specific antigenic peptides typically 8 to 18 amino acids in length play a critical role in both adoptive and adaptive immunotherapy strategies (211). Modified neoantigen peptides are initially degraded by the proteasome and subsequently transported into the endoplasmic reticulum (ER) by the transporter associated with antigen processing (TAP). Within the ER, these peptides bind to major MHC molecules, forming peptide–MHC (pMHC) complexes that are then presented on the surface of antigen-presenting cells (APCs). Upon presentation, neoepitopes displayed by MHC class I and II molecules engage TCRs on CD8^+^ and CD4^+^ T cells, respectively, thereby initiating their activation (212).

A thorough understanding of peptide–MHC and T-cell receptor interactions is essential for designing peptide-based cancer vaccines (213). Short peptides, usually composed of nine amino acids, can directly bind to MHC molecules. However, this direct binding may induce immune tolerance and facilitate rapid peptide degradation, posing challenges for effective vaccine development (214). Longer peptides, typically around 30 amino acids in length, are internalized and processed by APCs, allowing for presentation by both MHC class I and II molecules. This dual presentation activates CD8^+^ and CD4^+^ T cells, respectively, and supports the development of long-term immune memory, making long peptides potentially more effective at stimulating robust and durable antitumor immune responses (214). Neoantigens are well recognized as crucial targets for effective antitumor immunity (215). Multiple studies have demonstrated that a higher tumor neoantigen burden is associated with better clinical outcomes and stronger T-cell responses. Analysis of RNA-sequencing (RNA-seq) data from 18 solid tumor types in The Cancer Genome Atlas (TCGA) revealed a positive correlation between neoantigen frequency and the expression of genes linked to T-cell cytolytic activity (98). A study analyzing 515 tumors across six different histological subtypes from The Cancer Genome Atlas (TCGA) found that a higher burden of predicted immunogenic epitopes was significantly associated with improved patient survival (216). Whole-exome sequencing of 619 colorectal tumors in these studies revealed a positive association between high neoantigen burden and extended patient survival, as well as increased infiltration of tumor-infiltrating lymphocytes (TILs) (217). Furthermore, studies have shown a correlation between tumor-infiltrating lymphocyte (TIL) density and neoantigen load across various malignancies, including endometrial cancer, suggesting that higher neoantigen burden may promote greater immune cell infiltration (218). Secondly, robust anticancer immunity is marked by the expansion of neoantigen-specific T cell populations. This phenomenon has been observed in NSCLC patients treated with pembrolizumab (an anti-PD-1 antibody), as well as in melanoma patients who showed favorable clinical responses to ipilimumab (an anti-CTLA-4 antibody) (219–221). Furthermore, in patients undergoing adoptive transfer of TILs, tumor regression mediated by both CD4^+^ and CD8^+^ T cells is specifically directed against neoantigens, underscoring the critical role of neoantigen recognition in effective antitumor responses (219, 222–224). Third, both preclinical animal studies and clinical investigations have shown that neoantigen-specific T lymphocytes exert cytolytic activity against tumor cells presenting altered peptides, leading to tumor regression. In transplantable chemically induced sarcoma and genetically engineered sarcoma models expressing immunodominant antigens, CD8^+^ T cells recognized epitopes corresponding to neoantigens in rejected tumors (225, 226).

Immunization with neoantigen peptides elicited T cell responses that enhanced antitumor activity in both preventive and therapeutic settings within transplantable mouse models of colon cancer and melanoma (227–229). In a chemically induced sarcoma mouse model, vaccination with long neoantigen peptides capable of activating both CD4^+^ and CD8^+^ T cell responses induced tumor rejection comparable to that achieved with immune checkpoint inhibitor therapy (24). Neoantigen vaccines composed of MHC class I/II-restricted neoepitopes elicited strong tumor-specific immune responses and effectively induced rejection of colon cancer and melanoma xenografts in mice when delivered as poly-neoepitope mRNA formulations (230). Therapeutic neoantigens have shown significant potential across various preclinical animal models. Notably, adoptive transfer of neoepitope-specific CD4^+^ T cells induced tumor regression in a cholangiocarcinoma patient, providing direct clinical evidence of the anticancer effectiveness of neoantigen-specific T cells (231). Therefore, neoantigens are prime targets for therapeutic cancer vaccines and T cell-based adoptive cell transfer (ACT) immunotherapies (232–234). Mutations acquired during tumor development generate neoantigens that contribute to intratumoral heterogeneity (ITH). This genetic diversity within tumors profoundly influences the efficacy of treatments such as ICIs and CAR-T cell therapies (235). Tumor heterogeneity results in a mass composed of diverse cell populations with distinct molecular profiles and variable treatment sensitivities. This diversity can manifest as spatial heterogeneity, uneven distribution of genetically distinct subclones within primary or metastatic sites, or as temporal heterogeneity, characterized by dynamic changes in cancer cell molecular expression over time (235).

### 3.7 Metabolic reprogramming agents

The metabolic landscape of the TME plays a crucial role in promoting resistance to ICIs in non-small cell lung cancer by regulating antitumor immunity (236). Tumors frequently establish a metabolically suppressive milieu by accumulating immunosuppressive metabolites and depriving effector immune cells of essential nutrients. A well-characterized example is the adenosine pathway, where elevated extracellular adenosine in the TME inhibits T cell and NK cell functions through activation of the adenosine A2A receptor (A2AR) (237).

Pharmacological inhibition of A2AR has demonstrated promise in preclinical models by restoring T cell activation and synergizing with PD-1 blockade. Clinical trials combining ICIs with A2AR antagonists, such as ciforadenant, are currently underway in patients with advanced non-small cell lung cancer. Additionally, arginase inhibitors can counteract the immunosuppressive effects of myeloid cells that deplete arginine, a critical amino acid for T cell activation and proliferation, thereby enhancing T cell function and ICI efficacy through arginine restoration. Glutamate antagonists like CB-839 (telaglenastat), which target tumor glutamine metabolism, have also been shown to inhibit tumor growth and modulate immune cell activation within the tumor microenvironment. Collectively, these metabolic reprogramming agents offer innovative and practical strategies to boost antitumor immunity and overcome ICI resistance in metabolically hostile NSCLC tumors (**Table 4**) (238). In conclusion, targeting metabolic reprogramming, including adenosine signaling, arginase activity, and glutamine metabolism, offers a promising avenue to reverse immunosuppression and overcome ICI resistance specifically in NSCLC.

**Table 4:** Pharmacological strategies to overcome immune checkpoint inhibitor resistance in non-small cell lung cancer targets, agents, clinical insights, advantages, limitations, experimental outcomes, and key references.

Strategy	Target/mechanism	Key agents/examples	Clinical insights	Advantages	Disadvantages	Experimental Result	References
Targeting Alternative Immune Checkpoints	Non-redundant checkpoints increased in PD-1-resistant cancers include LAG-3, TIGIT, TIM-3, and VISTA.	LAG-3 Relatlimab + Nivolumab + TIGIT Tiragolumab + Atezolizumab + TIM-3 Sabatolimab The VISTA antagonists	Several treatments are in current studies; combined blockage may revitalize worn-out T cells and restore ICI effectiveness.	Increases immunological activation; can overcome adaptive resistance; and often works in concert with other ICIs.	An increase in unfavorable occurrences linked to the immune system Clinical benefit is still being studied; choosing the right biomarker is necessary.	NSCLC studies are still being conducted (NCT04623775); the RELATIVITY-047 study showed that Relatlimab + Nivolumab increased PFS (10.1 vs. 4.6 months) in melanoma compared to Nivolumab alone.	(239)
Modulating the Tumor Microenvironment (TME)	focusing on metabolic remodeling, immunological suppressive signaling, cytokine/chemokine axis, and TAMs	CSF-1R inhibitors; inhibition of CCR2/CCL2 Inhibitors of TGF-β IDO and arginase inhibitors	TAM depletion or reprogramming can improve T cell infiltration, alleviate immunosuppression, and raise ICI sensitivity.	Transforms “cold” tumors into “hot” ones Improves immune cell activity and access; it can be used in conjunction with other immunotherapies.	TME interactions are complex, and they might have unintended consequences. - Limited clinical effectiveness thus far	IMmotion trials: CSF-1R inhibitors in NSCLC had poor monotherapy activity but enhanced response with ICIs; TGF-β blocker + anti-PD-L1 in RCC showed increased CD8+ T cell infiltration (NCT02452424).	(240, 241)
Combining ICIs with Oncogenic Pathway Inhibitors	The MAPK pathway, EGFR, and KRAS (G12C) mutations cause immunological exclusion and resistance.	TKIs for EGFR (Osimertinib) Inhibitors of KRAS G12C (Sotorasib) Trametinib is an MEK inhibitor.	Effective in particular molecular subsets; sequencing techniques are necessary to reduce the risk of harm; potential synergy in trials.	A focused strategy for specific genotypes Oncogene-driven immune resistance may be reversed.	ICIs have a high risk of harm; only patients with actionable mutations are eligible. Resistance mutations might appear.	CodeBreaK100: In KRAS G12C NSCLC, sotorasib demonstrated a 37% ORR; this combination with anti-PD-1 is being studied (NCT04185883); preliminary findings indicate improved response but higher liver toxicity.	(242, 243)
Epigenetic Modulators	Reversing T cell fatigue with HDAC inhibition and DNA methylation	NCT02638090, NCT02437136, Vorinostat, Entinostat (HDACi), and Azacitidine (DNMTi) are the trials.	In resistant NSCLC, epigenetic treatment rewires the TME, improves antigen presentation, and reestablishes the ICI response.	Restoring immunological response, working in concert with ICIs, and perhaps having a wide range of applications	Off-target effects and toxicity Precise dose and timing are necessary; patient response varies.	NCT01928576: In preclinical NSCLC models, entinostat with anti-PD-1 boosted CD8+ T cell infiltration and MHC-I expression; in clinical trials, disease stability was reported in about 30% of patients.	(201, 202)
Cytokine-Based Strategies	Modification of IL-2 signaling to promote effector cells over Tregs	Pegylated/fused IL-2 variations; medicines targeted to the IL-2 receptor subunit	The goal of engineered IL-2 therapy is to reduce Treg activation and increase cytotoxic T cells specifically.	Enhances T cell activity and proliferation; modified forms lessen toxicity.	A limited window for therapy Thorough patient monitoring is necessary because systemic cytokine effects can be harmful.	Newer IL-2 variations are being developed. Bempegaldesleukin (NKTR-214) showed promise in conjunction with nivolumab (30% ORR in P1/2 studies); however, it did not enhance outcomes in P3 PIVOT IO-001 (no OS/PFS improvement).	(244–246)
Adoptive Cell Therapies and Vaccines	Peptide vaccines and T cells that target neoantigens improve tumor selectivity.	Long/short peptide neoantigen vaccinations Adoptive TIL transfer; neoepitope vaccines based on mRNA	A better ICI response is correlated with a higher neoantigen burden; TILs and vaccinations exhibit tumor-specific cytolytic activity.	Extremely individualized and particular; potential for long-lasting immunological memory Some experiments have shown encouraging outcomes.	Costly and logistically challenging; constrained by the load of tumor mutations Prolonged production	When used in conjunction with nivolumab, the NEO-PV-01 vaccination (NCT02897765) demonstrated improved neoantigen-specific T cell responses in NSCLC; In melanoma, adoptive TIL transfer produced a 36% ORR; NSCLC studies are still continuing (NCT04614103).	(247–254)

## 4 Translational lessons from targeted therapy: bridging molecular and immune resistance in NSCLC

**Figure 5:**
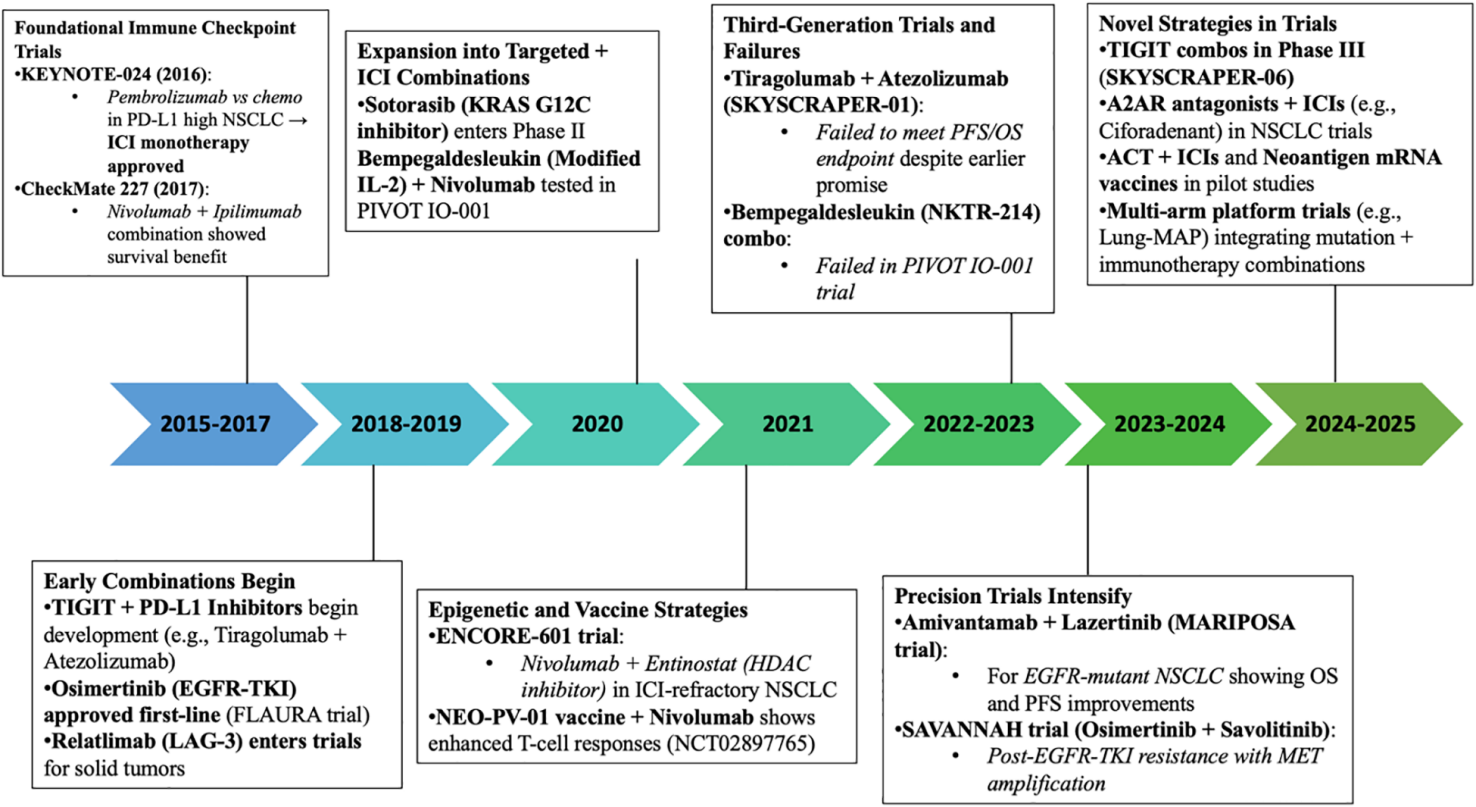
Timeline of Emerging Combination Therapies in NSCLC (2015–2025). A chronological overview of major clinical trials and therapeutic milestones in non-small cell lung cancer (NSCLC) that combine immune checkpoint inhibitors (ICIs) with other pharmacological strategies. The timeline highlights key developments including PD-1/PD-L1 monotherapy approvals (e.g., KEYNOTE-024, CheckMate 227) (13, 14), targeted therapy–ICI combinations (Sotorasib + anti-PD-1), epigenetic modulators (e.g., Entinostat, Azacitidine), cytokine-based agents (e.g., modified IL-2), TIGIT and LAG-3 checkpoint combinations (e.g., Tiragolumab, Relatlimab), as well as experimental approaches such as adoptive cell therapies (ACT), neoantigen vaccines, and metabolic reprogramming agents. This timeline reflects the shift toward biomarker-guided, precision immunotherapy and the dynamic evolution of strategies to overcome resistance in NSCLC.

The evolution of targeted therapies in NSCLC has provided crucial mechanistic insights that directly inform emerging strategies to overcome immune resistance. While inhibitors directed against EGFR, ALK, RET, and KRAS mutations have achieved remarkable clinical success, nearly all patients eventually experience relapse driven by adaptive processes such as secondary mutations, pathway reactivation, metabolic rewiring, or lineage plasticity. These mechanisms of molecular escape closely mirror the immune-evasion pathways observed following checkpoint blockade, revealing a convergent biology of resistance that links oncogenic signaling to immune suppression. As summarized in **Table 5**, the developmental trajectories of major targeted agents exhibit a consistent pattern of early, pronounced responses followed by acquired resistance through molecular adaptation. **Figure 5** further illustrates how the therapeutic landscape of NSCLC has evolved over the past decade from single-pathway inhibition to multidimensional regimens that integrate oncogenic blockade with immune modulation. Collectively, these translational milestones demonstrate how the principles derived from targeted-therapy resistance are now guiding the rational design of next-generation precision immunotherapy combinations aimed at converting immune-cold tumors into durable immune-responsive states.

**Table 5:** NSCLC clinical trials & resistance landscape.

Targeted pathway	Representative agent(s)	Dominant resistance mechanism	Relevance to immunotherapy resistance	Reference
EGFR	Osimertinib	C797S mutation, MET amplification	Demonstrates adaptive signaling and immune exclusion; supports combined EGFR + ICI strategies	(263)
RET	Selpercatinib, Pralsetinib	RET solvent-front mutations	Highlights the importance of targeting escape clones; conceptually parallels T-cell exhaustion.	(182)
MET	Capmatinib, Savolitinib	MET amplification	Links oncogenic bypass with myeloid-driven immunosuppression	(264)
KRAS G12C	Sotorasib, Adagrasib	KEAP1/STK11 co-mutations	Defines “immune-cold” phenotype; dual ICI combinations show emerging benefit	(56)
HER2/ALK	Trastuzumab deruxtecan, Lorlatinib	Secondary mutations, lineage switching	Suggests parallel adaptive mechanisms between oncogenic and immune resistance	(182)

### 4.1 EGFR-mutant NSCLC & osimertinib: a flagship success with emerging resistance

#### 4.1.1 Success

Osimertinib (Tagrisso^®^), a third-generation EGFR tyrosine kinase inhibitor (TKI), emerged in response to the notorious T790M “gatekeeper” mutation, the predominant resistance mechanism to first- and second-generation EGFR TKIs. In the phase III AURA3 trial, osimertinib more than doubled progression-free survival (PFS), 10.1 vs 4.4 months, compared to platinum-pemetrexed chemotherapy (HR 0.30) in T790M-positive NSCLC, leading to its FDA approval in 2017 (255–257). Subsequent FLAURA data confirmed that first-line osimertinib not only extended PFS and overall survival versus earlier TKIs, but also demonstrated superior central nervous system (CNS) penetration, crucial given the brain-metastasis proclivity in EGFR-driven disease (257).

#### 4.1.2 Resistance

Despite potent activity, resistance is nearly universal within approximately 10 months. The most common mechanisms observed in the AURA3 study include the on-target EGFR C797S mutation and MET amplification, each accounting for about 18% of cases (256). Additional routes include HER2 or PIK3CA amplification, RET and NTRK fusions, RAS-MAPK pathway activation, and histologic transformation to small-cell lung cancer (257). Loss of T790M can predict early progression, while C797S emergence often marks later relapse (257). Such resistance underscores the heterogeneous nature of underlying mechanisms observed in real-world NSCLC cohorts treated with osimertinib, emphasizing the urgent need for biomarker-guided sequencing and rational combination strategies to delay or overcome therapeutic resistance.

#### 4.1.3 Overcoming resistance

Prospective and real-world evidence support combining EGFR-targeted agents with other pathway inhibitors. For example, gefitinib plus capmatinib showed ~27% objective response rate (ORR) and PFS around 8 months in MET-amplified, EGFR-mutant NSCLC (256). Ongoing trials such as FLAURA-2 and MARIPOSA suggest that upfront combinations (osimertinib + chemotherapy or amivantamab + lazertinib) may delay or prevent resistance (255).

### 4.2 RET-fusion NSCLC: from multikinase modesty to selective efficacy

#### 4.2.1 Initial setback

Early RET targeting via multikinase inhibitors (MKIs) like cabozantinib and vandetanib delivered modest response rates (≤16%) and short PFS (~7 months) (258, 259).

#### 4.2.2 Major breakthrough

Selective RET inhibitors revolutionized outcomes in RET-fusion NSCLC (~1–2% of cases). Two agents stand out:

- Selpercatinib (Retevmo^®^): In the LIBRETTO-001 trial, the ORR reached 64–84% depending on prior treatment history, with durable responses (median DOR ~17.5 months) and notable intracranial activity (260).
- Pralsetinib (Gavreto^®^): The ARROW trial reported 57% ORR in previously treated patients; real-world case series mirrored these results with strong responses and manageable toxicities (261).

Both drugs received accelerated FDA approval in 2020, with selpercatinib later receiving full approval in 2024 following more mature data.

#### 4.2.3 Resistance

Like osimertinib, selective RET inhibitors encounter acquired resistance, typically through point mutations (e.g., RET G810 solvent-front substitutions), MET amplification, or KRAS activation (262). Sequential regimens or combination approaches await exploration.

### 4.3 Unified lessons from targeted NSCLC therapies

1. Driver-specific targeting yields rapid responses, but resistance inevitably emerges.
2. Resistance is heterogeneous mutational changes within the target (e.g., C797S, RET G810) or activation of bypass tracks (MET, KRAS, HER2).
3. Biomarker-informed sequencing and combination regimens are essential to prolong benefit and manage resistance.
4. Early selective combination therapies (e.g., osimertinib + chemotherapy or amivantamab) may delay resistance onset.
5. Continuous molecular profiling via tissue or plasma is critical to identify resistance mechanisms and guide next-line treatments.
6. Strategic trial design considering prior therapy, timing of drug use, control arms, and CNS endpoints can resurrect drugs (e.g., dacomitinib flourished in first-line settings after earlier failures) and bring treatments like selpercatinib and pralsetinib to fruition.

In summary, NSCLC precision oncology exemplifies both the power and limitations of targeted therapy: dramatic initial responses that are almost always followed by adaptive resistance. As molecular testing becomes routine and combination strategies mature, the field is shifting from drug-by-drug breakthroughs to integrated, evolving treatment algorithms designed not just to strike but to anticipate cancer’s countermoves. The next frontier lies in multitargeted regimens and smarter sequencing, guided by real-time biomarker tracking.

## 5 Challenges and future perspectives

Treating advanced NSCLC effectively requires not only potent therapies but also advances in predictive technologies and personalized strategies. One of the principal challenges lies in improving predictive biomarkers, the tools that anticipate which patients will respond to which treatments, particularly when resistance emerges. Although PD-L1 expression and TMB are clinically established for guiding ICIs, their predictive accuracy is imperfect. PD-L1 assays are hampered by variability in thresholds and tumor heterogeneity, while TMB measurement is subject to platform differences and lacks universally accepted cutoffs (265). Several emerging biomarkers, such as tertiary lymphoid structures (TLS), TILs, IFNγ gene signatures, circulating tumor DNA, and specific epigenetic patterns, hold promise to enhance prediction beyond these two standard markers (266). Importantly, these biomarkers are now being actively integrated into clinical trial design and therapeutic decision-making. Contemporary NSCLC trials increasingly incorporate PD-L1 expression (122), TMB, and genomic co-mutations (e.g., STK11, KEAP1) as key stratification variables for patient enrollment and treatment arms. For instance, multi-arm adaptive trials such as Lung-MAP and CheckMate 9LA apply biomarker-based randomization to tailor combination regimens (122), while ongoing phase II–III studies evaluate microbiome modulation or epigenetic reprogramming as response-enhancing strategies. In clinical practice, biomarker panels derived from tumor tissue or liquid biopsy now guide ICI selection, sequencing with targeted therapy, and management of resistance. This integration underscores the transition from population-level treatment paradigms to individualized, biomarker-driven decision-making in NSCLC immunotherapy (267).

Equally promising is the exploration of the microbiome as a predictive tool. Studies have shown that gut microbial diversity and specific taxa correlate with better responses to ICI therapy in NSCLC, suggesting that microbiome profiling or even modulation via probiotics or fecal transplant may improve clinical outcomes (268). However, findings are inconsistent across studies, highlighting the need for standardized methods and larger validation cohorts. Beyond biomarkers, the effective stratification of patients remains a key challenge (267). Integrating tumor genetics (e.g., EGFR, ALK, RET mutations, TMB), immune profiling (PD-L1, TILs, inflammatory signatures), and microbiome data could enable more precise grouping of patients with shared biology and risk profiles. Advanced frameworks like the HOHMS paradigm and liquid biopsy technologies (e.g., EPIC-Seq) support such integrative stratification by combining histologic, molecular, and circulating markers in real time (267).

As therapeutic complexity grows, combination therapies such as EGFR-TKI + MET inhibitors or ICI + chemotherapy + VEGF blockade offer new hope but also raise concerns about additive toxicities. Immune-related adverse events (irAEs) and pharmacogenomic sensitivity (e.g., via DPYD or UGT1A1 polymorphisms) can cause serious harm in susceptible individuals. Thus, companion diagnostics (“toxgnostics”) that flag high-risk patients before treatment are increasingly necessary (269). These challenges underscore the urgent need for personalized therapeutic approaches tailored to each patient’s unique molecular and immunologic profile. This requires shifting from population-based regimens to dynamic, adaptable treatment plans guided by ongoing monitoring (e.g., liquid biopsies via cfDNA or EPIC-Seq), biomarker-adjusted doses, and evolving combination schemes (269).

Despite these advances, combination regimens still face significant limitations. Multi-agent immunotherapy and targeted therapy often lead to cumulative toxicities and higher rates of irAEs, limiting tolerability and long-term efficacy. Moreover, many strategies lack validated biomarkers to guide patient selection, and current assays remain inconsistent across trials (270). These challenges highlight the need for standardized biomarker validation frameworks, integration of safety-focused companion diagnostics (“toxgnostics”), and adaptive monitoring systems within future combination designs (270).

Looking ahead, the path to fuller precision in NSCLC lies in:

- Biomarker refinement and validation: Creating standardized, reproducible assays for predictive markers, genetic, immune, and microbial to guide frontline and resistance therapy.
- Multidimensional stratification: Integrating genomics, immune states, and microbiome profiles to identify patient subgroups with shared therapeutic vulnerabilities.
- Adaptive trial design: Embedding real-time biomarker monitoring and response-driven treatment adjustments into trials, rather than fixed pipelines.
- Safety-focused personalization: Developing companion toxicological tests to detect patients susceptible to severe immune or drug toxicities, enabling preemptive dose adjustment or alternative regimens.
- Personalized dynamic therapy algorithms: Tracking patient profiles over time using liquid biopsy and adjusting combinations or sequences of targeted agents, immunotherapies, and microbiome modifiers to outpace evolving resistance.

This vision marks a critical transformation in NSCLC treatment: moving from one-size-fits-all interventions toward individualized, data-driven cancer care, designed to anticipate resistance, maximize efficacy, and minimize harm (271). However, significant gaps remain in understanding the temporal dynamics of resistance evolution, cross-talk between oncogenic and immune pathways, and optimal sequencing of combination regimens, areas that warrant continued translational and clinical investigation (271).

The rapid evolution of computational oncology has opened new frontiers in addressing therapeutic resistance. Among these innovations, artificial intelligence (AI) stands out as a transformative force, capable of decoding the immense biological complexity underlying ICI resistance in NSCLC (182). By integrating data from genomics, transcriptomics, radiomics, and clinical outcomes, AI provides multidimensional insight into how tumor-intrinsic and extrinsic mechanisms evolve during therapy (272). Machine learning (ML) and deep learning (DL) frameworks can identify subtle molecular signatures that predict resistance, monitor treatment dynamics through liquid biopsy and imaging data, and optimize combination regimens in silico before clinical application (273). Furthermore, AI-driven drug discovery and multi-omics integration are accelerating the design of next-generation immunomodulators targeting pathways such as LAG-3, VISTA, IDO1, and A2AR. These advances mark a paradigm shift from empirical treatment selection toward data-driven precision immunotherapy, laying the foundation for the next stage of progress, discussed in Section 5.1 (122).

### 5.1 Role of artificial intelligence in overcoming ICI resistance

The advent of artificial intelligence (AI) has revolutionized the field of oncology, offering innovative approaches to address the complex challenge of ICI resistance in NSCLC. ICI therapies, targeting pathways such as PD-1/PD-L1 and CTLA-4, have transformed NSCLC treatment, yet primary, acquired, and adaptive resistance mechanisms limit their efficacy in many patients. These mechanisms, driven by tumor-intrinsic factors (e.g., neoantigen loss, defective antigen presentation), tumor-extrinsic factors (e.g., immunosuppressive TME) (274), and immune escape, necessitate novel strategies to predict, monitor, and overcome resistance. AI, encompassing machine learning (ML), deep learning (DL), and computational modeling, provides powerful tools to analyze multidimensional data (275), identify resistance mechanisms, optimize therapeutic strategies, and personalize treatment plans. By integrating genomic, proteomic, imaging, and clinical data, AI enhances our understanding of ICI resistance and supports the development of combination therapies to improve outcomes in NSCLC (276).

AI’s role in overcoming ICI resistance begins with predictive modeling to identify patients likely to develop resistance. ML algorithms, such as random forests and neural networks, can analyze genomic and transcriptomic data from tumor biopsies to predict primary resistance (277). For instance, AI models trained on datasets from NSCLC patients treated with anti-PD-1 therapies can identify signatures associated with low neoantigen burden or defective interferon-gamma (IFN-γ) signaling, key tumor-intrinsic resistance mechanisms (278). These models integrate features like TMB, PD-L1 expression, and MHC class I downregulation to stratify patients into responders and non-responders (279). Studies have demonstrated that AI-driven biomarkers, such as those derived from RNA sequencing or single-cell transcriptomics, achieve higher predictive accuracy than traditional biomarkers like PD-L1 immunohistochemistry alone (279). By identifying patients at risk of primary resistance before treatment initiation, AI enables clinicians to tailor first-line therapies, potentially combining ICIs with targeted agents or TME-modulating drugs to preempt resistance. Beyond prediction, AI facilitates real-time monitoring of acquired resistance during ICI therapy (141). Longitudinal analysis of circulating tumor DNA (ctDNA) and immune cell profiles using liquid biopsies provides dynamic insights into tumor evolution and immune escape. DL models, particularly convolutional neural networks (CNNs), can process high-dimensional ctDNA sequencing data to detect emerging mutations associated with resistance, such as loss of heterozygosity in MHC genes or upregulation of alternative checkpoints like TIM-3 or LAG-3 (272). Similarly, AI can analyze flow cytometry or single-cell RNA sequencing data to monitor shifts in TME composition, such as increased infiltration of MDSCs or Tregs, which drive adaptive resistance. These AI-driven approaches enable early detection of resistance, allowing timely adjustments to treatment regimens, such as switching to combination therapies targeting immunosuppressive pathways. For example, AI models identifying TME-driven resistance could guide the use of adenosine pathway inhibitors or TGF-β blockers, which are under investigation in NSCLC trials (141, 272, 279).

AI also plays a critical role in optimizing combination therapies to overcome ICI resistance. The complexity of the TME, characterized by interactions between immune cells, tumor cells, and mediators like arginase-1, adenosine, and VEGF, requires integrative approaches to design effective regimens (277). AI-driven computational models, such as agent-based or systems biology models, simulate TME dynamics to predict the impact of combining ICIs with other therapies. For instance, ML algorithms can analyze preclinical and clinical trial data to identify synergistic combinations, such as anti-PD-1 with anti-TIGIT or EGFR inhibitors like Osimertinib (278). These models incorporate variables like drug pharmacokinetics, immune cell activation, and cytokine profiles to optimize dosing schedules and minimize toxicity (279). In NSCLC, AI has been used to prioritize combinations like sotorasib (KRAS G12C inhibitor) with anti-PD-1 in patients with KRAS-mutant tumors, based on predictive models of tumor-immune interactions (279). By simulating therapeutic outcomes, AI reduces the reliance on trial-and-error approaches, accelerating the development of effective combination strategies (280).

Radiomics, a subset of AI, enhances the assessment of ICI resistance through imaging analysis. DL algorithms applied to computed tomography (CT) or positron emission tomography (PET) scans can extract quantitative features, such as tumor texture, size, and spatial heterogeneity, to predict resistance patterns (272). For example, radiomic signatures from baseline CT scans have been correlated with TME immunosuppression, such as high TAM infiltration or low CD8^+^ T cell density, which are associated with primary resistance. Longitudinal radiomic analysis can also detect subtle changes in tumor morphology indicative of acquired resistance, complementing ctDNA and immune profiling (281). In NSCLC, studies have shown that radiomic models outperform traditional RECIST criteria in predicting ICI response, offering a non-invasive method to guide treatment decisions. By integrating radiomic data with genomic and clinical variables, AI creates comprehensive predictive models that enhance precision medicine approaches. AI-driven drug discovery is another promising avenue for overcoming ICI resistance (282). Computational platforms using generative adversarial networks (GANs) or reinforcement learning can design novel molecules targeting resistance mechanisms, such as alternative checkpoints (e.g., VISTA, LAG-3) or metabolic pathways (e.g., IDO1, A2AR) (283). These platforms analyze large chemical libraries and protein structures to identify candidates with high binding affinity and specificity. In NSCLC, AI has accelerated the development of small-molecule inhibitors for immunosuppressive TME mediators, such as arginase-1 inhibitors, which enhance T-cell function. Additionally, AI-guided neoantigen discovery supports the development of personalized vaccines and adoptive cell therapies (283). By analyzing tumor exome sequencing data, AI identifies immunogenic neoantigens likely to elicit strong T-cell responses, improving the efficacy of vaccines or TCR-engineered therapies (284). Clinical trials combining neoantigen vaccines with ICIs in NSCLC are leveraging AI to optimize antigen selection, demonstrating improved response rates in early-phase studies (283).

The integration of AI with multi-omics data further enhances its impact on ICI resistance. Multi-omics approaches, combining genomics, proteomics, metabolomics, and transcriptomics, provide a holistic view of tumor-immune interactions. AI algorithms, such as graph neural networks, can integrate these datasets to uncover novel resistance pathways, such as epigenetic silencing of antigen-presenting genes or metabolic reprogramming via adenosine production (285). In NSCLC, AI-driven multi-omics analysis has identified biomarkers like STK11/LKB1 mutations, which are associated with an immunosuppressive TME and poor ICI response. These insights guide the development of targeted interventions, such as combining ICIs with epigenetic modulators like HDAC inhibitors to restore antigen presentation (285). By synthesizing multi-omics data, AI enables a systems-level understanding of resistance, facilitating the design of personalized treatment plans. Despite its potential, AI implementation faces challenges, including data quality, interpretability, and generalizability (272, 285, 286). High-quality, standardized datasets are essential for training robust AI models, yet NSCLC datasets often vary in format and completeness. Interpretability of DL models, often described as “black boxes, “ remains a hurdle for clinical adoption, necessitating explainable AI frameworks to build clinician trust (281, 282). Additionally, AI models trained on specific cohorts may not generalize across diverse NSCLC populations, requiring validation in global trials. Addressing these challenges through collaborative data-sharing initiatives and transparent model development will enhance AI’s clinical utility (141, 278). In conclusion, AI is transforming the approach to overcoming ICI resistance in NSCLC by enabling predictive modeling, real-time monitoring, optimized combination therapies, radiomic analysis, and novel drug discovery. By integrating multi-omics, imaging, and clinical data, AI uncovers resistance mechanisms and tailors interventions to individual patients (276, 277). In summary, the most promising strategies to overcome ICI resistance in NSCLC include rational combination regimens that integrate ICIs with targeted, epigenetic, and metabolic modulators; biomarker-driven patient stratification guided by multi-omic profiling; and the application of artificial intelligence to predict, monitor, and personalize treatment responses. Together, these multidisciplinary approaches offer a path toward durable clinical benefit and the realization of true precision immunotherapy in NSCLC (182, 287).

## 6 Conclusion

The emergence of immune checkpoint inhibitors (ICIs) has transformed the therapeutic landscape of NSCLC, yet resistance remains a dominant clinical challenge. The most promising strategies to overcome this barrier lie in rational combination therapies that integrate ICIs with targeted, epigenetic, and metabolic modulators to restore immune activity and counter adaptive escape. Parallel advances in adoptive cell therapies, neoantigen vaccines, and cytokine engineering are expanding the therapeutic arsenal beyond traditional checkpoints. Equally important, biomarker-driven precision medicine guided by genomic, proteomic, and microbiome profiling will enable more effective patient stratification and therapy sequencing. Artificial intelligence and multi-omic integration now provide the tools to predict resistance, monitor dynamic tumor evolution, and personalize intervention in real time. Collectively, these multidisciplinary innovations mark a transition from empirical immunotherapy to data-driven, precision immuno-oncology, paving the way for durable and individualized clinical benefit in NSCLC.
